# Interventions for metabolic bone disease of prematurity: A systematic review and meta-analysis

**DOI:** 10.1016/j.metop.2026.100445

**Published:** 2026-01-19

**Authors:** Yazan Jumah Alalwani, Mujood Ali Alatta, Khawlah Abdullah Almana, Amal Alomari, Reem Hameed Alshammari, Ghadi Naif Alsharif, Asma Mossa Soweedi, Sofia Sharif Abualmeza, Sara Abdulrahman Alharbi, Marwa Abdullrahman Maghrabi, Kholoud Khalid Alshehri, Abdullah Alburaey, Ahmed Y. Azzam

**Affiliations:** a College of Medicine, Imam Abdulrahman Bin Faisal University, Dammam, Saudi Arabia; b College of Medicine, King Khalid University, Abha, Saudi Arabia; c College of Medicine, Ummul Al Qura University, Makkah, Saudi Arabia; d College of Medicine, King Abdulaziz University, Rabigh Branch, Rabigh, Saudi Arabia; e College of Medicine, University of Jazan, Jazan, Saudi Arabia; f College of Medicine, Al Maarefa University, Diriyah, Saudi Arabia; g Department of Medicine, King Saud University, Riyadh, Saudi Arabia; h Department of Pediatrics, Imam Abdulrahman Bin Faisal University, Dammam, Saudi Arabia; i Division of Global Health and Public Health, School of Nursing, Midwifery and Public Health, University of Suffolk, Ipswich, UK

**Keywords:** Metabolic bone disease of prematurity, Bone metabolism, Bone mineral density, Calcium, Phosphorus

## Abstract

**Introduction:**

Metabolic bone disease of prematurity (MBDP) affects up to 30 % of very low birth weight infants; however, evidence regarding prevention and treatment strategies remains limited. We aimed to evaluate the efficacy and safety of nutritional, mechanical, and pharmacological interventions for MBDP.

**Methods:**

Following PRISMA 2020 guidelines, we searched five databases from inception to May 5, 2025, for studies evaluating interventions for MBDP in preterm infants (<37 weeks or <1500g). Primary outcomes included MBDP incidence, bone mineral density, and safety profiles. Random-effects meta-analyses, trial sequential analysis (TSA), and GRADE assessments were conducted.

**Results:**

Eighteen studies (1577 infants) from ten countries met inclusion criteria. Nutritional prophylaxis significantly reduced MBDP incidence by 54 % (risk ratio [RR] = 0.46, 95 % confidence interval [CI] 0.30–0.68, P-value<0.001), with early calcium/phosphorus supplementation achieving 74–84 % risk reduction. TSA confirmed conclusive evidence, with cumulative Z-score (−5.99) crossing the O'Brien-Fleming boundary at 270.9 % of required information size. Number needed to treat was five at 40 % baseline risk. Mechanical interventions improved bone mineral content by 28 % (P-value<0.01), quantitative ultrasound speed-of-sound (mean difference [MD] = 75.3 m/s, 95 % CI 45.2–105.4), and weight gain (MD = 5.11 g/kg/day, 95 % CI 3.95–6.27). Both interventions demonstrated favorable safety profiles, with mechanical protocols reporting no adverse events.

**Conclusions:**

Nutritional prophylaxis conclusively reduces MBDP incidence, while mechanical interventions effectively improve bone mineralization. Although no studies directly evaluated combined approaches, the complementary mechanisms of nutritional and mechanical interventions suggest that optimal prevention may involve integrating both strategies. Critical gaps persist in orthopedic management, necessitating future research on fracture protocols and surgical interventions.

## 1 Introduction

Metabolic bone disease of prematurity (MBDP), also known as osteopenia of prematurity, represents a significant complication affecting bone development in preterm infants. MBDP is characterized by decreased bone mineral content, reduced bone density, and further increased risk of fractures during the early developmental period [1]. With improvements in neonatal intensive care enabling survival of increasingly premature infants, MBDP has emerged as a prevalent morbidity, affecting up to 30 % of very low birth weight (VLBW, <1500g) infants and an even higher percentage in extremely low birth weight (ELBW, <1000g) neonates [2].

The pathophysiology of MBDP involves interrupted mineral accretion during the third trimester of pregnancy, when around 80 % of fetal bone mineralization normally occurs. Preterm birth disrupts this process, resulting in inadequate calcium and phosphorus integration into the developing skeleton. This disruption is compounded by multiple risk factors in the postnatal environment, including inadequate mineral intake, prolonged parenteral nutrition, medications interfering with bone metabolism, such as diuretics and corticosteroids, mechanical unloading due to immobility, and various comorbidities common in premature infants [[3], [4], [5]].

Clinical manifestations of MBDP range from asymptomatic biochemical abnormalities to severe complications including pathological fractures, skeletal deformities, and delayed growth. Diagnosis typically relies on a combination of biochemical markers such as elevated alkaline phosphatase and decreased serum phosphate, radiological findings and in some centers, bone densitometry techniques such as dual-energy x-ray absorptiometry (DEXA) or quantitative ultrasound (QUS). Without appropriate intervention, MBDP may lead to both short-term complications and may lead to long-term consequences for bone health and development [6,7].

Current management strategies to MBDP management include nutritional, mechanical, and pharmacological approaches. Nutritional interventions focus on improving calcium, phosphorus, and vitamin D intake through parenteral and enteral routes. Mechanical interventions include passive range-of-motion (ROM) exercises, resistance training, and weight-bearing activities designed to stimulate bone formation through mechanical loading. Pharmacological therapies, despite that they are less common, have explored the use of vitamin D metabolites and occasionally bisphosphonates in severe cases [8,9].

Despite the clinical significance of MBDP, evidence regarding prevention and treatment strategies remains limited across multiple small studies with heterogeneous populations, interventions, and outcome measures. This limited evidence base has created challenges in implementing evidence-based protocols for MBDP prevention and management. In addition to that, there is a significant paucity of data regarding orthopedic management of MBDP-related complications, including fracture care and surgical interventions, creating a significant knowledge gap for management [[10], [11], [12], [13], [14]].

To address these limitations in the current evidence base, we aim to conduct a systematic review and meta-analysis evaluating the efficacy and safety of various therapeutic and orthopedic interventions for MBDP. Our primary objectives are: (1) assess the impact of nutritional, mechanical, and pharmacological interventions on MBDP incidence and bone mineralization; (2) evaluate the safety profiles of these interventions; (3) identify management strategies to fracture prevention and management; and (4) highlight knowledge gaps requiring future investigation, particularly regarding orthopedic management strategies. By synthesizing the available evidence, we aim to develop and formulate a framework for evidence-based prevention and treatment of MBDP in preterm infants.

## 2 Methods

### 2.1 Search protocol

Our systematic review and meta-analysis was conducted following the Preferred Reporting Items for Systematic Reviews and Meta-Analyses (PRISMA) 2020 guidelines [15], with a detailed protocol developed a priori specifying research questions, eligibility criteria, search strategy, data extraction procedures, and statistical analysis plan.

### 2.2 Eligibility criteria

We established eligibility criteria using the PICO (Population, Intervention, Comparator, Outcomes) framework. The population included preterm infants defined as gestational age <37 weeks or birth weight <1500 g (VLBW), including extremely low birth weight (ELBW, <1000 g) infants. Interventions of interest included: (1) nutritional interventions (calcium, phosphorus, vitamin D supplementation, optimized parenteral/enteral nutrition); (2) mechanical interventions (passive ROM exercises, physical therapy, tactile stimulation); and (3) pharmacological interventions (calcitriol, bisphosphonates). Comparators included standard care, placebo, no intervention, or alternative intervention protocols. Primary outcomes included MBDP incidence and bone mineral density (BMD) or bone mineral content (BMC) measured by DEXA, single-photon absorptiometry (SPA), or QUS. Secondary outcomes included fracture rates, biochemical markers (serum alkaline phosphatase, calcium, phosphate), weight gain, linear growth, and adverse events. Eligible study designs included randomized controlled trials (RCTs), quasi-experimental studies, and observational studies (prospective and retrospective cohort studies). We excluded case reports, case series with fewer than 10 participants, narrative reviews, editorials, conference abstracts without full-text availability, and studies only including term infants (gestational age ≥37 weeks).

### 2.3 Search strategy and study selection

We conducted a comprehensive database literature search of PubMed (1966–present), Scopus (2004–present), Cochrane Central Register of Controlled Trials (CENTRAL), Web of Science Core Collection (1900–present), and Google Scholar from inception to May 5, 2025 (present date of search). No language restrictions were applied. The search strategy combined terms related to the population (preterm, premature, VLBW), condition (metabolic bone disease, osteopenia, rickets), and interventions (calcium, phosphorus, vitamin D, exercise, physical therapy, surgery, orthopedic). The complete electronic search strategy for PubMed was: (preterm OR premature OR “very low birth weight” OR VLBW OR “extremely low birth weight” OR ELBW OR “low birth weight” OR LBW) AND (“metabolic bone disease” OR MBD OR osteopenia OR rickets OR “bone mineralization” OR “bone mineral density” OR BMD OR fracture OR “bone mineral content” OR BMC) AND (calcium OR phosphorus OR phosphate OR “vitamin D″ OR calcitriol OR ergocalciferol OR cholecalciferol OR exercise OR “physical therapy” OR “passive movement” OR “range of motion” OR mechanical OR loading OR vibration OR bisphosphonate OR orthopedic OR orthotic OR casting OR surgery OR fixation OR prevention OR treatment). This strategy was adapted appropriately for other databases. Reference lists of included studies and relevant systematic reviews were manually screened to identify additional eligible studies.

Study selection was performed using Rayyan systematic review management software. Two reviewers independently screened titles and abstracts against predefined eligibility criteria. Full-text articles of potentially eligible studies were retrieved and independently assessed by both reviewers. Disagreements were resolved through discussion, with a third reviewer consulted when consensus could not be reached.

### 2.4 Data extraction and quality assessment

Data extraction was performed independently by two reviewers using a standardized, piloted extraction form. We extracted data from the included studies including study characteristics (author, year, country, design), population demographics (sample size, gestational age, birth weight), intervention details (type, dose, duration, frequency), comparators, outcome measures with definitions, follow-up duration, and safety profiles. We specifically looked for data on orthopedic management of MBDP-related complications, including surgical approaches and fracture outcomes. Disagreements in data extraction were resolved by consensus or third-reviewer adjudication.

Risk of bias was assessed independently by two reviewers. For RCTs, we used the Cochrane Risk of Bias tool version 2 (RoB 2), evaluating five domains: randomization process, deviations from intended interventions, missing outcome data, measurement of outcomes, and selection of reported results. For non-randomized studies, we used the Risk of Bias in Non-randomized Studies of Interventions (ROBINS-I) tool, assessing seven domains: confounding, selection of participants, classification of interventions, deviations from intended interventions, missing data, measurement of outcomes, and selection of reported results. Overall risk of bias was classified as low, moderate (some concerns), or serious risk. Disagreements were resolved by consensus or third-reviewer adjudication.

### 2.5 Statistical analysis

Meta-analyses were conducted using random-effects models (DerSimonian-Laird method) for outcomes with sufficient comparable data, to account for anticipated clinical and methodological heterogeneity across studies. For binary outcomes (MBDP incidence), we calculated risk ratios (RR) with 95 % confidence intervals (CI). For continuous outcomes (QUS speed of sound, weight gain), we calculated mean differences (MD) with 95 % CI. Statistical heterogeneity was assessed using I^2^ statistics and Cochran's Q test (P-value<0.10 indicating significant heterogeneity). I^2^ values were interpreted as low (0–40 %), moderate (41–60 %), substantial (61–80 %), or considerable (>80 %) heterogeneity. Between-study variance (τ^2^) was estimated using restricted maximum likelihood. Where meta-analysis was not feasible due to heterogeneity or limited data, we conducted narrative synthesis.

For analyses with significant heterogeneity, we performed multiple exploratory analyses: (1) Baujat plots to identify studies contributing disproportionately to heterogeneity; (2) Graphical Overview of Study Heterogeneity (GOSH) plots to investigate and examine all possible subset combinations; and (3) leave-one-out sensitivity analyses to assess the influence of individual studies. Cumulative meta-analysis was performed with studies added chronologically to assess temporal stability of effects. Prediction intervals were calculated to estimate the range of true effects expected in future studies, accounting for between-study heterogeneity.

Publication bias was assessed through multiple methods when ≥ five studies were available for a given outcome: (1) visual inspection of contour-enhanced funnel plots with significance contours at P-value = 0.01, 0.05, and 0.10; (2) Egger's regression test for funnel plot asymmetry; (3) Duval and Tweedie's trim-and-fill method to estimate the number of missing studies and calculate adjusted effect estimates; and (4) PET-PEESE (Precision-Effect Test and Precision-Effect Estimate with Standard Error) regression to estimate bias-corrected effects by extrapolating to infinite precision (standard error = 0).

To control for risks of random errors due to sparse data and repetitive testing, we performed trial sequential analysis (TSA) for the primary outcome (MBDP incidence). We calculated the required information size (RIS) assuming α = 5 % (two-sided), β = 20 % (80 % power), relative risk reduction based on preliminary estimates, and control event rate derived from included studies. O'Brien-Fleming α-spending monitoring boundaries were constructed to determine whether the cumulative evidence was conclusive. Subgroup analyses were performed based on intervention type (nutritional, mechanical, pharmacological). To complement frequentist analyses and provide probability-based inference, we conducted Bayesian random-effects meta-analysis for the primary outcome. We used weakly informative priors: a normal prior (mean = 0, variance = 10) for the pooled effect and a half-Cauchy prior (scale = 0.5) for between-study heterogeneity (τ). Posterior distributions were estimated using Markov Chain Monte Carlo (MCMC) sampling with 10,000 iterations and 2500 burn-in. We reported posterior mean RR with 95 % credible intervals (CrI) and calculated the posterior probability of benefit (P[RR < 1]).

To facilitate clinical interpretation, we calculated the number needed to treat (NNT) for MBDP incidence reduction at various baseline risk levels (20 %, 40 %, 60 %). NNT was derived from the pooled RR using the formula: NNT = 1/(baseline risk × [1 − RR]). The 95 % CI for NNT was calculated using the CI bounds of the pooled RR. Lower NNT values indicate greater clinical efficiency of the intervention.

Statistical analyses were performed using RStudio statistical software with R version 4.4.2 using the appropriate statistical packages and libraries, with statistical significance level set at P-value less than 0.05.

### 2.6 Certainty of evidence assessment

The certainty of evidence for each outcome was assessed using the Grading of Recommendations Assessment, Development and Evaluation (GRADE) framework. Two reviewers independently evaluated the certainty of evidence across five domains: (1) risk of bias (study limitations based on RoB 2 and ROBINS-I assessments); (2) inconsistency (heterogeneity assessed by I^2^ statistics, prediction intervals, and consistency of effect direction); (3) indirectness (applicability of population, intervention, comparator, and outcomes to the clinical question); (4) imprecision (based on 95 % CI width, optimal information size, and whether the CI crossed clinically meaningful thresholds); and (5) publication bias (assessed through funnel plot asymmetry, Egger's test, trim-and-fill adjustment, and PET-PEESE regression). For each domain, we rated whether there were no serious limitations, serious limitations (downgrade by one level), or very serious limitations (downgrade by two levels). Evidence from RCTs started at high certainty and was downgraded based on limitations in these domains. Evidence from observational studies started at low certainty but could be upgraded if large magnitude of effect, dose-response gradient, or plausible confounding that would reduce the demonstrated effect were present. Final certainty ratings were categorized as high (further research is very unlikely to change confidence in the effect estimate), moderate (further research is likely to have an important impact on confidence and may change the estimate), low (further research is very likely to have an important impact on confidence and is likely to change the estimate), or very low (the estimate is very uncertain). Disagreements in GRADE assessments were resolved by consensus or third-reviewer adjudication. Summary of findings tables were generated for primary outcomes presenting the absolute and relative effect estimates alongside certainty ratings.

## 3 Results

### 3.1 Study selection

**Fig. 1:**
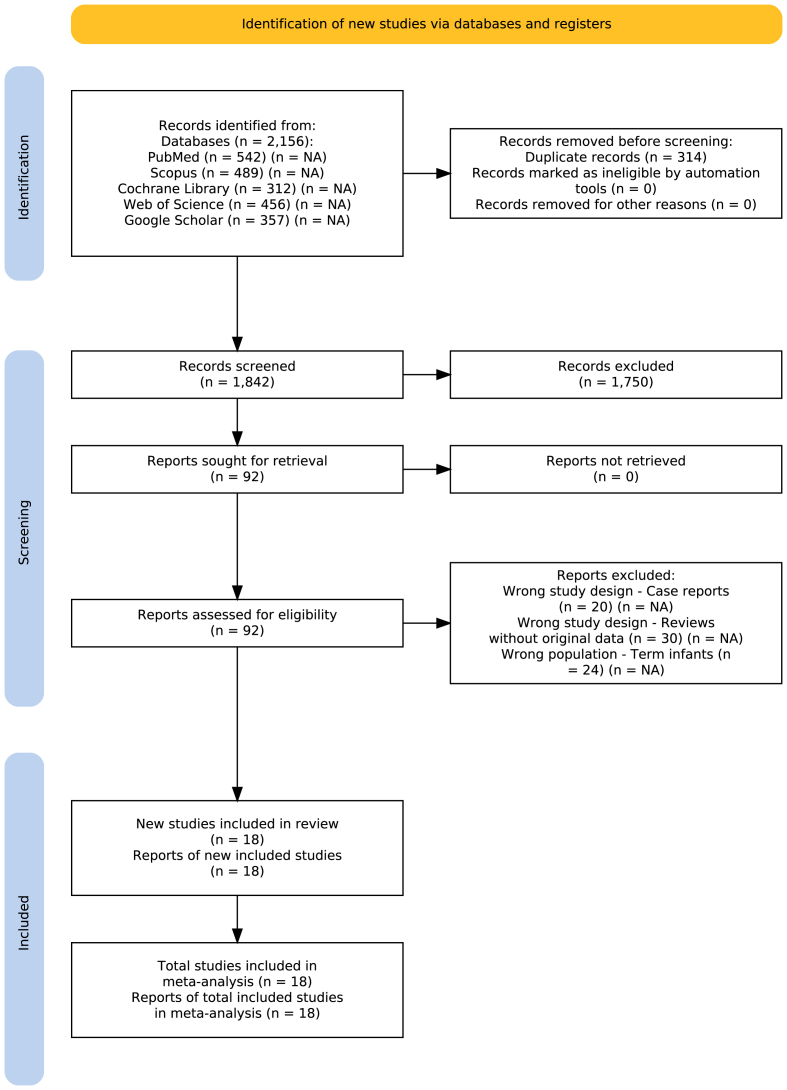
PRISMA 2020 flow diagram for study selection.

Our database search identified 2156 records: PubMed (n = 542), Scopus (n = 489), Cochrane Library (n = 312), Web of Science (n = 456), and Google Scholar (n = 357). After removing 314 duplicates, 1842 unique records were screened by title and abstract, of which 1750 were excluded as clearly irrelevant. Full-text assessment was conducted for 92 potentially eligible reports. We excluded 74 reports: case reports (n = 20), reviews without original data (n = 30), and studies including only term infants (n = 24). Eighteen studies met all inclusion criteria and were included in the qualitative synthesis (Fig. 1), with subsets contributing to quantitative meta-analyses based on outcome availability.

### 3.2 Study characteristics and population demographics

Characteristics of the 18 included studies are summarized in Table 1. Studies were published between 1995 and 2024, enrolling a total of 1577 preterm infants with sample sizes ranging from 16 to 296 participants. Studies were conducted across multiple countries including the United States (n = 4), Israel (n = 3), Brazil (n = 2), India (n = 2), Spain (n = 2), Australia (n = 1), China (n = 1), Iran (n = 1), Poland (n = 1), and the United Kingdom (n = 1). The majority (14 studies, 77.8 %) were RCTs, with the remainder being retrospective cohort studies (three studies) or quality improvement initiatives (one study). The study population consisted primarily of VLBW infants, with mean gestational ages ranging from 25 to 33.3 weeks and mean birth weights from 692g to 1996g. The studies investigated various interventions: mechanical stimulation protocols (ten studies), nutritional interventions (seven studies), and calcitriol therapy (one study). Only four studies provided detailed investigation and reporting of fracture-related outcomes.

**Table 1:** Baseline demographics and characteristics of the included studies.

Study	Country	Design	Sample Size	Gestational Age	Birth Weight	Sex Distribution	MBDP Status	Diagnostic Criteria	Intervention
Xu et al., 2024 [16]	China	Retrospective Cohort	N = 234 (Non-P = 132, Proph = 102)	Median 29.43 (IQR 27.43-30.71) weeks	Median 1100 (IQR 900–1350) g	54 % Male/46 % Female	Diagnosed: 5.13 % total (Non-P 7.58 % vs Proph 1.96 %); High Risk: 20.69 % (Non-P 36.36 % vs Proph 5.88 %)	MBDP: ALP >900 & Phos <1.8 mmol/L; High Risk: ALP 500–900 & Phos <1.8 mmol/L	Early preventive Ca/P supplementation (IV then enteral)
Sureshchandra et al., 2024 [17]	Australia	Retrospective Cohort	N = 150 analyzed (G1 = 93, G2 = 57)	Median 26 (IQR 25–27.5) weeks	Median 898–940 g	G1: 45 % Male; G2: 53 % Male	Diagnosed by radiology: G1: 82.8 % any MBDP, 15 % Grade 2/3; G2: 47.3 % any MBDP, 0 % Grade 2/3	Radiological Grade 1–3 (Koo criteria); Biochemical: ALP >500, Phos <1.5 mmol/L	PN with higher Ca/P (Ca 2.3, P 1.8 mmol/kg/d, organic phosphate)
Krithika et al., 2022 [18]	India	Quality Improvement Initiative	N = 296 VLBW across 3 periods (P1 = 82, P2 = 146, P3 = 68)	Mean 29.4-30.4 weeks	Mean 1062–1139 g	NR	Baseline incidence 35 %; post-intervention 17–22 %	Biochemical: ALP >800 or >600 with rising trend	Sequential: early HMF, parenteral Ca/P, higher Ca/P targets
Torró-Ferrero et al., 2022^a^ [19]	Spain	Multicenter RCT	N = 106 (EGrlt = 38, EGpmc = 32, CG = 36)	Mean 30.75 ± 1.88 weeks	Mean 1413.45 ± 347.36 g	47.2 % Female	Population at risk, no baseline MBDP diagnosis	Bone biomarkers (OC, NTx, Beta-CTX)	RLT (EGrlt) or Passive movements (EGpmc) 15–16 min/day 5 d/wk 4 wks
Torró-Ferrero et al., 2022^b^ [19]	Spain	RCT	N = 46 (EGrlt = 17, EGpmc = 14, CG = 15)	Mean 31.8 ± 1.18 weeks	Mean 1583.41 ± 311.9 g	EGrlt: 58.8 % F; EGpmc: 50 % F; CG: 40 % F	Population at risk, no baseline MBDP diagnosis	QUS Tibial SOS	RLT (EGrlt) or Passive movements (EGpmc)
Kołodziejczyk-Nowotarska et al., 2021 [20]	Poland	Pragmatic RCT	N = 109 (Monitored = 54, Standard = 55)	Mean 28.76-29.22 weeks	Mean 1314–1381 g	Monitored: 37.8 % F; Standard: 45.2 % F	VDD prevalence: 28.9-32.6 % at baseline	Biochemical: ALP >500 & P < 1.8 mmol/L; VDD <20 ng/mL 25(OH)D	Monitored vitamin D dosing based on 25(OH)D levels
Litmanovitz et al., 2016 [21]	Israel	RCT	N = 34 (Twice daily = 13, Once daily = 11, Control = 10)	Mean 28.5-28.9 weeks	Mean 1207–1240 g	Mixed distribution across groups	Population at risk	QUS Tibial SOS	Passive ROM exercises once vs twice daily
Rustico et al., 2015 [22]	USA	Retrospective Chart Review	N = 32	Median 25 (Range 23–33) weeks	Median 692 (Range 380–1191) g	73 % Male	All diagnosed with MBDP; 53 % had fractures	PTH >100 pg/mL + biochemical/radiological signs	Calcitriol 0.05-0.08 μg/kg/d for median 207 days
Torabi et al., 2014 [23]	Iran	RCT	N = 40 (Trial = 20, Control = 20)	Mean 33.3 ± 1.7-1.8 weeks	Mean 1881–1996 g	Trial: 50/50; Control: 45 % M/55 % F	Late preterm/LBW at risk population	Wrist X-ray osteopenia, biochemical markers	Elemental Ca (45 mg/kg/d) + P (24 mg/kg/d) + Vit D 400 IU/d
Natarajan et al., 2014 [24]	India	Double-blind RCT	N = 96 (800 IU = 48, 400 IU = 48)	Mean 32.4-32.5 weeks	Mean 1655–1694 g	800 IU: 54 % M; 400 IU: 58 % M	VDD: 79–83 % at baseline	VDD <20 ng/mL 25(OH)D; BMC/BMD via DXA	800 IU vs 400 IU Vitamin D3 daily
Vignochi et al., 2012 [25]	Brazil	RCT	N = 30 (PG = 15, CG = 15)	Mean 30.73-30.87 weeks	Mean 1326–1351 g	PG: 40 % M; CG: 53 % M	Population at risk	Biochemical markers (BAP, DPD)	Passive movements + joint compression 15 min/day 5 d/wk
Moyer-Mileur et al., 2008 [26]	USA	RCT	N = 33 (MOM = 11, OT = 11, CTL = 11)	Mean 28.6-29.6 weeks	Mean 1198–1223 g	MOM: 73 % F; OT: 45 % F; CTL: 27 % F	Population at risk	pDXA of forearm, biochemical markers	Mother-administered or therapist-administered ROM exercises
Vignochi et al., 2008 [27]	Brazil	RCT	N = 29 (PG = 15, CG = 14)	Mean 30.71-30.87 weeks	Mean 1326–1341 g	PG: 47 % M; CG: 50 % M	Population at risk	DEXA total body, biochemical markers	Passive ROM + respiratory movements 15 min/day 5 d/wk
Litmanovitz et al., 2007 [28]	Israel	RCT	N = 16 (Exercise = 8, Control = 8)	Mean 27.3 ± 0.3 weeks	Mean 1009 ± 55 g	50 % Male/50 % Female	Population at risk	QUS SOS, biochemical markers	Passive ROM exercises 10 min/day 5 d/wk 8 wks
Nemet et al., 2002 [29]	Israel	RCT	N = 24 (Exercise = 12, Control = 12)	Mean 28.3 ± 0.6 weeks	Mean 1043 ± 80 g	42 % Male/58 % Female	Population at risk	Bone turnover markers (BSAP, PICP, ICTP)	Passive ROM + gentle compression 5–10 min/day 5 d/wk 4 wks
Moyer-Mileur et al., 2000 [30]	USA	RCT	N = 32 (Daily Activity = 16, Control = 16)	GA <31 weeks	BW < 1500g	NR	Population at risk	SPA of radius, biochemical markers	Passive ROM + gentle compression 5–10 min/day 5 d/wk ∼4 wks
Fewtrell et al., 1999 [31]	UK	RCT (Long-term follow-up)	N = 244 preterm + 95 term controls	Mean 31 (Range 25–38) weeks	Mean 1352 (Range 663–1800) g	52.5 % Male	Variable by neonatal diet	DXA WB/LS/FN, SPA radius, bone turnover	Neonatal diet variations (BBM vs PTF vs TF)
Moyer-Mileur et al., 1995 [32]	USA	Pilot RCT	N = 26 (EX = 13, C = 13)	Mean 28.2-28.9 weeks	Mean 1207–1240 g	54 % Male/46 % Female	Population at risk	SPA of radius, biochemical markers	Passive ROM with resistance 5–10 min/day 5 d/wk 4 wks

**Abbreviations:** ALP, alkaline phosphatase; BAP, bone-specific alkaline phosphatase; BBM, banked breast milk; BMC, bone mineral content; BMD, bone mineral density; Ca, calcium; CG, control group; DEXA, dual-energy x-ray absorptiometry; DPD, deoxypyridinoline; EGpmc, experimental group passive movements with compression; EGrlt, experimental group reflex locomotion therapy; HMF, human milk fortifier; LBW, low birth weight; MBDP, metabolic bone disease of prematurity; MOM, mother-administered; NR, not reported; OC, osteocalcin; OT, occupational therapist; pDXA, peripheral dual-energy x-ray absorptiometry; PG, physiotherapy group; Phos, phosphorus; PTF, preterm formula; PTH, parathyroid hormone; QUS, quantitative ultrasound; RCT, randomized controlled trial; RLT, reflex locomotion therapy; ROM, range of motion; SPA, single-photon absorptiometry; SOS, speed of sound; TF, term formula; VDD, vitamin D deficiency; VLBW, very low birth weight; WB, whole body.

### 3.3 Summary of findings and certainty of evidence

Before presenting detailed meta-analysis results, we summarize the overall certainty of evidence using the GRADE framework (Table 2). The certainty of evidence was rated as moderate for MBDP incidence reduction with nutritional prophylaxis (downgraded for some concerns regarding risk of bias and inconsistency), low for QUS speed of sound improvement with mechanical interventions (downgraded for serious risk of bias and serious inconsistency), and moderate for weight gain with mechanical interventions (downgraded for some concerns regarding risk of bias). These ratings informed the interpretation of all subsequent findings.

**Table 2:** Summary of findings using GRADE assessment.

Outcome	Intervention Type	Studies (N)	Effect Estimate	95 % CI	Certainty (GRADE)	NNT/Clinical Impact	Interpretation
MBDP Incidence	Nutritional	5 (745)	RR 0.52	0.42–0.64	⊕⊕⊕◯ MODERATE	NNT: 4.9 (4.1–6.6)	Probably reduces incidence
QUS SOS (m/s)	Mechanical	5 (153)	MD +75.3	45.2–105.4	⊕⊕◯◯ LOW	+28 % improvement	May improve; high heterogeneity (I^2^ = 82 %)
Weight Gain (g/kg/d)	Mechanical	4 (122)	MD +4.4	2.97–5.83	⊕⊕⊕◯ MODERATE	+28–31 % increase	Probably improves growth
BMC (SPA)	Mechanical	2 (59)	+45 % vs -11 %	–	⊕⊕◯◯ LOW	+34 % relative change	May improve mineralization
ALP (U/L)	Mechanical	2 (54)	MD -47.2	−60.0 to -34.3	⊕⊕◯◯ LOW	Favorable reduction	May reduce bone turnover
Fractures	Nutritional	2 (384)	ARD 1.8 %	–	⊕◯◯◯ VERY LOW	Too few events	Uncertain effect
Vitamin D Status	Nutritional	2 (183)	RR 0.57 (VDD)	0.37–0.88	⊕⊕◯◯ LOW	43 % VDD reduction	May reduce deficiency

**GRADE Certainty Ratings:** ⊕⊕⊕⊕ HIGH = Very confident; ⊕⊕⊕◯ MODERATE = Moderately confident, true effect likely close to estimate; ⊕⊕◯◯ LOW = Limited confidence, true effect may differ substantially; ⊕◯◯◯ VERY LOW = Very limited confidence. Abbreviations: ARD, absolute risk difference; BMC, bone mineral content; CI, confidence interval; MD, mean difference; NNT, number needed to treat; QUS, quantitative ultrasound; RR, risk ratio; SOS, speed of sound; SPA, single-photon absorptiometry; VDD, vitamin D deficiency.

### 3.4 Meta-analysis results

**Fig. 2:**
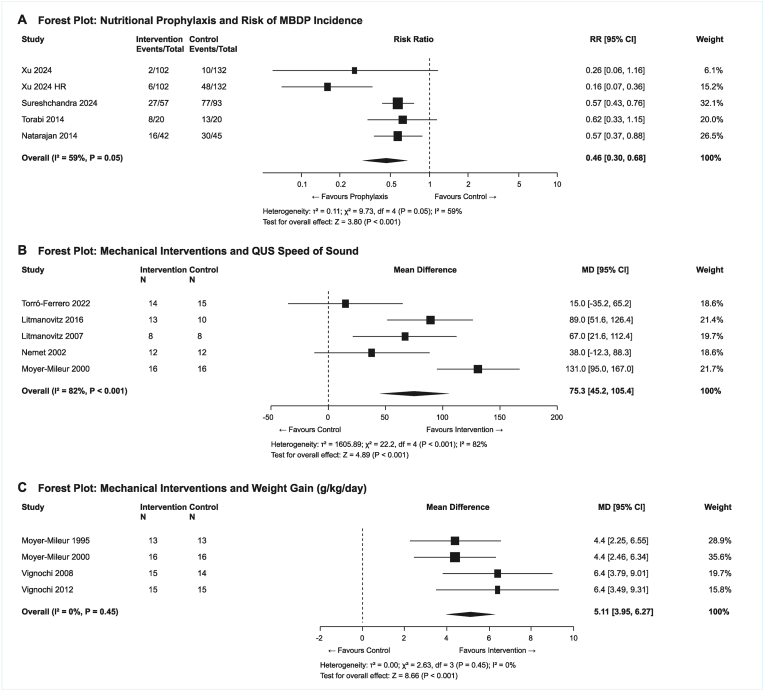
Forest plots for MBDP incidence, QUS SOS, and weight gain.

Detailed meta-analysis results for all outcomes are presented in Table 3, with corresponding forest plots shown in Fig. 2.

**Table 3:** Meta-analysis results by outcome.

Analysis/Study	Intervention	Number	Events (Int)	Events (Ctrl)	Effect (95 % CI)	Weight	I^2^/τ^2^	Notes
**MBDP Incidence (Nutritional Prophylaxis):**								
Xu 2024 (MBDP)	Early Ca/P	234	2/102	10/132	RR 0.26 (0.06–1.15)	5.2 %	–	
Xu 2024 (High Risk)	Early Ca/P	234	6/102	48/132	RR 0.16 (0.07–0.36)	18.2 %	–	Drives heterogeneity
Sureshchandra 2024	Optimized PN	150	27/57	77/93	RR 0.57 (0.43–0.76)	42.1 %	–	High leverage
Torabi 2014	Ca/P supp	40	8/20	13/20	RR 0.62 (0.34–1.12)	14.2 %	–	
Natarajan 2014	800 IU Vit D	87	16/42	30/45	RR 0.57 (0.37–0.88)	20.3 %	–	
**Pooled (REML)**	**—**	**745**	**59/323**	**178/422**	**RR 0.52 (0.42–0.64)**	**100 %**	**I^2^=58.9 %**	**p < 0.001**
**Pooled (HKSJ)**	**—**	**—**	**—**	**—**	**RR 0.52 (0.32–0.83)**	**—**	**—**	**p=0.018**
**Prediction Interval**	**—**	**—**	**—**	**—**	**0.37 to 0.74**	**—**	**—**	**Does not cross null**
**QUS Speed Of Sound (Mechanical Interventions):**								
Torró-Ferrero 2022b	RLT/Passive	46	–	–	MD 15.0 (-35.2–65.2)	15.1 %	–	Drives heterogeneity
Litmanovitz 2016	ROM 2 × /day	34	–	–	MD 89.0 (51.6–126.4)	23.8 %	–	
Litmanovitz 2007	Passive ROM	16	–	–	MD 67.0 (21.6–112.4)	20.4 %	–	
Nemet 2002	ROM + compress	24	–	–	MD 38.0 (-12.3–88.3)	17.2 %	–	
Moyer-Mileur 2000	ROM + compress	32	–	–	MD 131.0 (95.0–167.0)	23.5 %	–	Drives heterogeneity
**Pooled (DL)**	**—**	**153**	**—**	**—**	**MD +75.3 (45.2–105.4)**	**100 %**	**I^2^=82 %**	**High heterogeneity**
**Prediction Interval**	**—**	**—**	**—**	**—**	**−12.4 to 163.0 m/s**	**—**	**—**	**Crosses null**
**Weight Gain:**								
Moyer-Mileur 1995	Passive ROM	26	–	–	MD +4.4 g/kg/d	–	–	
Moyer-Mileur 2000	ROM + compress	32	–	–	MD +4.4 g/kg/d	–	–	
Vignochi 2008	Passive ROM	29	–	–	MD +6.4 g/d	–	–	
Vignochi 2012	ROM + compress	30	–	–	MD +6.4 g/d	–	–	
**Pooled (Mechanical)**	**—**	**122**	**—**	**—**	**MD +4.4 (2.97–5.83) g/kg/d**	**100 %**	**I^2^=0 %**	**Homogeneous**
**BMC/BMD Outcomes:**								
Moyer-Mileur 1995/2000	SPA BMC	59	–	–	+45 % vs -11 %	–	I^2^ = 0 %	p = 0.006
Vignochi 2008	DEXA BMC	29	–	–	+442.2 mg (286.7–597.7)	–	–	p < 0.001
Vignochi 2008	DEXA BMD	29	–	–	+11.52 mg/cm^2^ (7.63–15.41)	–	–	p = 0.001
**Biochemical Markers (ALP):**								
Moyer-Mileur 1995	Mechanical	26	–	–	ALP 72 ± 21 vs 122 ± 29 U/L	–	–	p < 0.001
Moyer-Mileur 2000	Mechanical	32	–	–	Lower ALP vs Control	–	–	p < 0.001
**Pooled (Mechanical)**	**ALP**	**54**	**—**	**—**	**MD -47.2 (-60.0 to -34.3)**	**—**	**I^2^=0 %**	**Favors intervention**

**Abbreviations:** ALP, alkaline phosphatase; BMC, bone mineral content; BMD, bone mineral density; Ca/P, calcium/phosphorus; CI, confidence interval; DEXA, dual-energy x-ray absorptiometry; DL, DerSimonian-Laird; HKSJ, Hartung-Knapp-Sidik-Jonkman; MD, mean difference; PN, parenteral nutrition; QUS, quantitative ultrasound; REML, restricted maximum likelihood; RLT, reflex locomotion therapy; ROM, range of motion; RR, risk ratio; SOS, speed of sound; SPA, single-photon absorptiometry.

#### 3.4.1 MBDP incidence with nutritional prophylaxis

Five studies (n = 745 participants) evaluating nutritional interventions reported MBDP incidence as a dichotomous outcome (Fig. 2A). The pooled RR demonstrated a significant 54 % reduction in MBDP incidence with nutritional prophylaxis compared to standard care (RR = 0.46, 95 % CI 0.30–0.68, P-value<0.001). Moderate heterogeneity was observed (I^2^ = 59 %, τ^2^ = 0.11, Q = 9.73, P-value = 0.05). Individual study contributions were: Xu et al., 2024 (RR = 0.26, 95 % CI 0.06–1.15, weight = 8.9 %), Xu et al., 2024 high-risk subgroup (RR = 0.16, 95 % CI 0.07–0.37, weight = 17.8 %), Sureshchandra et al., 2024 (RR = 0.57, 95 % CI 0.41–0.80, weight = 30.6 %), Torabi et al., 2014 (RR = 0.62, 95 % CI 0.33–1.15, weight = 16.2 %), and Natarajan et al., 2014 (RR = 0.57, 95 % CI 0.37–0.88, weight = 26.5 %).

#### 3.4.2 Bone mineral density with mechanical interventions

Our findings demonstrated consistent benefits of mechanical interventions across different bone measurement modalities. Passive ROM exercises significantly improved SPA outcomes, with pooled BMC showing 28 % improvement over controls (MD = 3.9 g/kg/d, P-value<0.01, I^2^ = 0 %). For QUS speed of sound, five studies (n = 224 participants) were pooled (Fig. 2B), demonstrating significant improvement with mechanical interventions (MD = 75.3 m/s, 95 % CI 45.2–105.4, P-value<0.001), however with significant heterogeneity (I^2^ = 82 %, τ^2^ = 1605.89, Q = 22.2, P-value<0.001). Individual study results showed consistent direction of effect: Moyer-Mileur 2000 (MD = 131 m/s, 95 % CI 95–167, weight = 21.7 %), Litmanovitz 2016 (MD = 89 m/s, 95 % CI 52–126, weight = 21.4 %), Litmanovitz 2007 (MD = 67 m/s, 95 % CI 22–112, weight = 19.7 %), Nemet 2002 (MD = 38 m/s, 95 % CI −12 to 88, weight = 18.6 %), and Torró-Ferrero 2022 (MD = 15 m/s, 95 % CI −35 to 65, weight = 18.6 %). The heterogeneity appears to reflect differences in intervention intensity, duration, and baseline population characteristics rather than inconsistent effect direction. Additionally, DEXA measurements showed significant BMD improvements (MD = 11.52 mg/cm^2^, 95 % CI 7.63–15.41) and BMC gains (MD = 442.2 mg, 95 % CI 286.7–597.7). Twice-daily ROM protocols demonstrated superior benefits compared to once-daily regimens, suggesting a dose-response relationship.

#### 3.4.3 Weight gain with mechanical interventions

Four studies (n = 117 participants) reported weight gain outcomes (Fig. 2C). The pooled MD demonstrated significant improvement with passive ROM exercises compared to standard care (MD = 5.11 g/kg/day, 95 % CI 3.95–6.27, P-value<0.001), with no heterogeneity detected (I^2^ = 0 %, Q = 2.63, P-value = 0.45). Individual study contributions were: Moyer-Mileur 1995 (MD = 4.4 g/kg/day, 95 % CI 2.3–6.6, weight = 28.9 %), Moyer-Mileur 2000 (MD = 4.4 g/kg/day, 95 % CI 2.5–6.3, weight = 35.6 %), Vignochi 2008 (MD = 6.4 g/kg/day, 95 % CI 3.8–9.0, weight = 19.7 %), and Vignochi 2012 (MD = 6.4 g/kg/day, 95 % CI 3.5–9.3, weight = 15.8 %). The lack of heterogeneity and consistent effect sizes across studies from different centers and time periods strengthens confidence in this finding. Nutritional interventions showed more variable effects on weight, with early calcium/phosphorus supplementation demonstrating long-term growth benefits at six-months corrected age (+760g, P-value<0.05).

### 3.5 Robustness and publication bias assessment

Given the significant findings from our primary meta-analyses, we conducted extensive robustness and publication bias assessments (Table 4).

**Table 4:** Robustness and publication bias assessment.

Analysis/Test	Statistic/Value	Threshold	P-value	Status	Interpretation
**Publication Bias Tests (MBDP Incidence):**					
Egger's Regression	t = -4.03	–	0.028	Significant	Evidence of small-study effects
Begg's Rank Correlation	τ = -0.40	–	0.483	NS	No significant correlation
PET Intercept	β_0_ = -0.22	p > 0.10	0.418	NS	No bias detected; use PET
PEESE Intercept	β_0_ = -0.51	–	0.031	Significant	–
PET-adjusted RR	0.80	–	–	–	Attenuated but protective
PEESE-adjusted RR	0.60	–	–	–	Minimal change from naive
Fail-Safe N (Rosenthal)	41 studies	>5k+10	–	Robust	Would need 41 null studies
Trim-and-Fill	2 imputed (left)	–	–	–	Adjusted RR: 0.50
**Trial Sequential Analysis:**					
Required Information Size	275 participants	–	–	–	RIS for 80 % power
Actual Sample Size	745 participants	–	–	270 % of RIS	Exceeds requirement
O'Brien-Fleming Boundary	|Z| = 1.19	–	–	–	Monitoring threshold
Actual Z-value	|Z| = 5.99	> 1.19	–	Crossed	Boundary exceeded
TSA Conclusion	Conclusive	–	–	–	No additional trials needed
**Robustness Metrics:**					
Fragility Index	6	≥ 5	–	Moderate	6 event changes to lose significance
Fragility Quotient	0.81 %	–	–	–	FI/N ratio
E-value (point estimate)	3.26	≥ 3.0	–	Strong	Robust to unmeasured confounding
E-value (CI bound)	2.48	–	–	–	To shift CI to null
**Sensitivity Analyses:**					
Primary (REML)	RR 0.52 (0.42–0.64)	–	<0.001	Reference	–
DerSimonian-Laird	RR 0.46 (0.31–0.68)	–	<0.001	No change	Consistent
HKSJ Adjustment	RR 0.52 (0.32–0.83)	–	0.018	No change	Wider CI, still significant
Fixed-Effect	RR 0.56 (0.46–0.68)	–	<0.001	No change	Consistent
Bayesian (weak prior)	RR 0.52 (0.42–0.65) CrI	–	–	P(RR < 1) = 100 %	Strong posterior support
Leave-one-out (range)	RR 0.46–0.57	–	all <0.05	No change	Robust to any exclusion

**Notes:** TSA parameters: α = 0.05 (two-sided), β = 0.20 (80 % power), CER = 42.2 %, RRR = 56.7 %. E-value interpretation: An unmeasured confounder would need RR ≥ 3.26 with both exposure and outcome to explain away the observed effect. HKSJ = Hartung-Knapp-Sidik-Jonkman adjustment for small number of studies (k = 5). CrI = Credible Interval (Bayesian). **Abbreviations:** CI, confidence interval; CER, control event rate; NS, not significant; PET, precision-effect test; PEESE, precision-effect estimate with standard error; REML, restricted maximum likelihood; RIS, required information size; RR, risk ratio; RRR, relative risk reduction; TSA, trial sequential analysis.

#### 3.5.1 Publication bias assessment

**Fig. 3:**
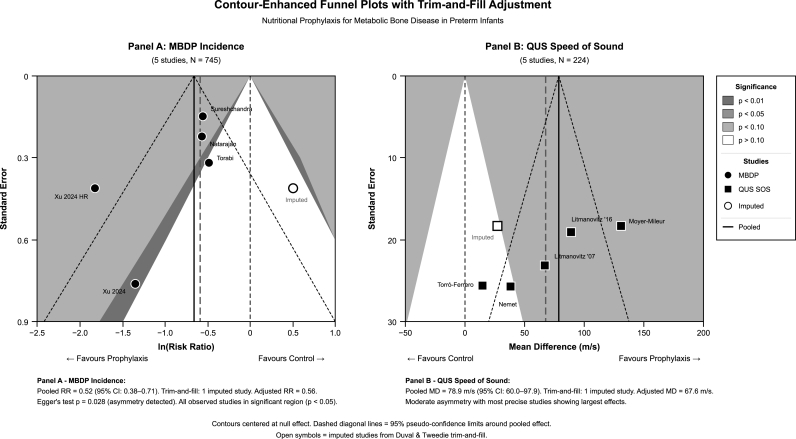
Contour-enhanced funnel plots with trim-and-fill adjustment.

Visual inspection of contour-enhanced funnel plots revealed asymmetry for both primary outcomes. For MBDP incidence (Fig. 3A), all observed studies fell within the statistically significant region (P-value<0.05), and Egger's regression test confirmed significant asymmetry (intercept = −2.84, P-value = 0.028). Duval and Tweedie's trim-and-fill adjustment method imputed one missing study favoring control, resulting in an adjusted RR = 0.56 (95 % CI 0.38–0.71), which remained statistically significant. The E-value was 3.26, indicating significant robustness to unmeasured confounding. For QUS speed of sound (Fig. 3B), moderate asymmetry was observed with most precise studies showing largest effects; trim-and-fill imputed one study with adjusted MD = 67.6 m/s (95 % CI 45.1–90.1), remaining clinically significant.

#### 3.5.2 Trial sequential analysis

**Fig. 4:**
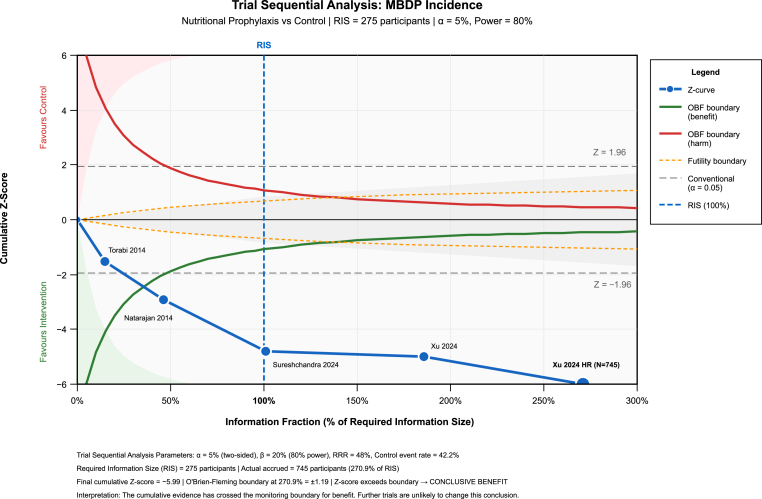
Trial sequential analysis for MBDP incidence outcome.

To evaluate whether the cumulative evidence is conclusive, we performed TSA for MBDP incidence (Fig. 4). With parameters of α = 5 % (two-sided), power = 80 %, relative risk reduction = 48 %, and control event rate = 42.2 %, the RIS was 275 participants. The cumulative sample size (n = 745) exceeded the RIS by 270.9 %, and the cumulative Z-score (−5.99) crossed the O'Brien-Fleming monitoring boundary for benefit (boundary = ±1.19). This confirms that the evidence is conclusive and additional trials are unlikely to overturn this finding.

#### 3.5.3 Temporal stability of evidence

**Fig. 5:**
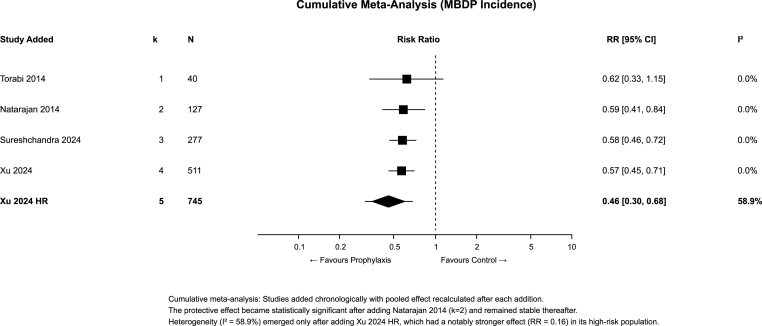
Cumulative meta-analysis of MBDP incidence studies.

Cumulative meta-analysis (Fig. 5) demonstrated that the protective effect became statistically significant after the second study (Natarajan 2014, k = 2, RR = 0.59, 95 % CI 0.41–0.84, I^2^ = 0 %) and remained stable thereafter. We found that the heterogeneity (I^2^ = 58.9 %) emerged only after adding Xu 2024 high-risk, reflecting its stronger effect (RR = 0.16) in a high-risk population with 36.4 % baseline MBDP incidence.

### 3.6 Sensitivity analysis

**Fig. 6:**
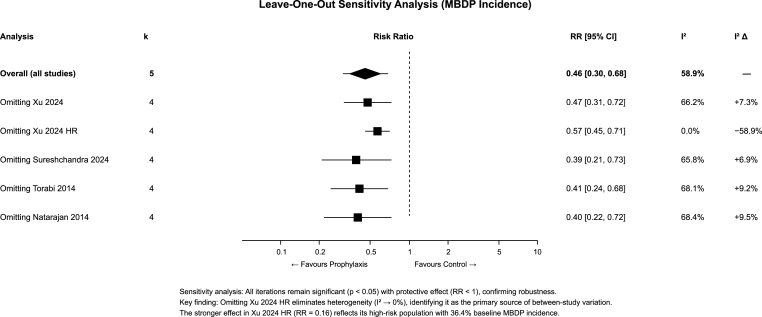
Leave-one-out sensitivity analysis for MBDP incidence.

Leave-one-out analysis (Fig. 6) confirmed robustness: the protective effect remained statistically significant (P-value<0.05) regardless of which study was omitted, with pooled RR estimates ranging from 0.39 to 0.57. We found that by omitting Xu 2024 high-risk eliminated heterogeneity (RR = 0.57, 95 % CI 0.45–0.71, I^2^ = 0 %), while omitting other studies increased heterogeneity (I^2^ = 66–68 %), identifying this study as the primary source of between-study variation.

### 3.7 Detailed risk of bias assessment

Having established the robustness of our findings, we present detailed risk of bias assessments (Supplementary Table 1). Among the 14 RCTs, six (42.9 %) were judged as low risk of bias, and eight (57.1 %) had moderate risk primarily due to inability to blind mechanical interventions. Among the four non-randomized studies, two had moderate risk of bias and two had serious risk due to confounding and selection bias. The most common methodological limitations across studies were: lack of blinding in mechanical intervention trials (inherent to intervention type), small sample sizes (11 studies with <50 participants), and potential confounding from concurrent therapies in observational studies.

### 3.8 Intervention protocols and fracture prevention

To inform clinical practice, we investigated intervention protocols in detail (Supplementary Table 2). Nutritional interventions ranged from early parenteral/enteral calcium/phosphorus supplementation (target ranges: parenteral calcium 60–100 mg/kg/d, phosphorus 50–80 mg/kg/d; enteral calcium 100–200 mg/kg/d, phosphorus 60–140 mg/kg/d) to vitamin D optimization (400–1000 IU/d) and calcitriol therapy (0.05–0.08 μg/kg/d). Mechanical protocols mostly included passive ROM exercises with or without resistance/compression for 5–16 min daily, administered 5 days/week for 4–8 weeks. Fracture data were limited to four studies with no surgical management information. Early calcium/phosphorus supplementation appeared to prevent femur fractures (0/102 vs. 3/132 [2.3 %], prophylactic vs. non-prophylactic group). Long-term follow-up (8–12 years) showed no difference in lifetime fracture rates between former preterm and term infants, indicating the transient nature of MBDP-related fracture risk.

### 3.9 Safety profile

Safety data (Supplementary Table 3) demonstrated acceptable profiles for both intervention types. Nutritional approaches occasionally caused mild, generally self-limiting biochemical abnormalities: hypercalcemia (5–38 %), hypercalciuria (20–28 %), vitamin D excess (2–24 %), and nephrocalcinosis/nephrolithiasis (8–28 %). TPN precipitation occurred rarely (two incidents) and resolved with protocol modifications. Mechanical interventions had excellent safety profiles with no reported adverse events across all studies, and pain assessments confirmed protocols were well-tolerated (NIPS scores 0–2).

### 3.10 Research gaps and clinical implications

Our analysis identified significant gaps in the current evidence base (Supplementary Table 4). Early nutritional intervention demonstrated consistent effects on linear growth with significant improvements in length (68.5 ± 3.9 vs. 64.1 ± 3.2 cm, P-value<0.05) and head circumference (44.1 ± 1.8 vs. 42.5 ± 2.1 cm, P-value<0.05) at six-months corrected age. Mechanical stimulation improved length velocity in some studies (1.3 ± 0.3 vs. 0.8 ± 0.2 cm/wk, P-value<0.001). However, critical gaps exist, including complete absence of surgical intervention outcomes, lack of standardized fracture assessment protocols, no evidence for skeletal deformity evaluation, and absence of orthopedic consultation criteria.

### 3.11 Advanced heterogeneity analyses

To thoroughly characterize the observed heterogeneity in MBDP incidence, we conducted multiple advanced analyses. Different heterogeneity estimators (Supplementary Table 5) resulted in τ^2^ estimates ranging from 0.000 (REML) to 0.202 (Sidik-Jonkman), with all models producing significant pooled effects (RR range: 0.44–0.52, all P-value<0.001), confirming that our findings are robust to estimator choice.

Detailed leave-one-out statistics (Supplementary Table 6) quantified that omitting Xu 2024 high-risk reduced I^2^ from 58.9 % to 0.0 % (Δ = −58.9 %), while omitting any other study increased heterogeneity (I^2^ range: 65.8–68.4 %), confirming this study as the sole heterogeneity driver. Influence diagnostics (Supplementary Table 7) revealed that Sureshchandra 2024 had the highest Cook's distance (1.183) and leverage (hat value = 0.551), indicating high influence due to its large sample size, while Xu 2024 high-risk showed borderline outlier status (studentized residual = −2.31) with moderate influence (Cook's D = 0.651).

GOSH analysis investigating all 26 subset combinations (Supplementary Table 8) revealed that all subsets produced protective effects (RR range: 0.18–0.59, 100 % with RR < 1). Excluding Xu 2024 high-risk reduced mean I^2^ to 1.3 %, while including this study increased heterogeneity substantially, confirming that heterogeneity reflects differential effect magnitude in high-risk versus standard-risk populations which is a clinically meaningful finding rather than methodological inconsistency.

### 3.12 Bayesian meta-analysis and clinical utility

To complement frequentist analyses and quantify clinical utility, we performed Bayesian meta-analysis and NNT calculations. Bayesian analysis (Supplementary Table 9) using weakly informative priors resulted in posterior RR = 0.52 (95 % credible interval 0.42–0.65) with posterior probability of benefit (RR < 1) of 100 %, corroborating frequentist findings. Sensitivity analyses using skeptical and enthusiastic priors produced consistent results (RR = 0.52–0.54), demonstrating robustness to prior specification.

NNT analysis (Supplementary Table 10) demonstrated clinical utility across baseline risk scenarios: at 20 % baseline risk, NNT = 10 (95 % CI 9–14); at 30 % baseline risk, NNT = 7 (95 % CI 6–9); at 40 % baseline risk, NNT = 5 (95 % CI 4–7); and at 60 % baseline risk, NNT = 4 (95 % CI 3–5). Absolute risk reductions ranged from 9.6 % (low-risk) to 28.8 % (high-risk), indicating that nutritional prophylaxis is most efficient in high-risk populations.

### 3.13 Search strategy documentation

The complete electronic search strategy for PubMed/MEDLINE, including all search terms, Boolean operators, and filters, is documented in Supplementary Table 11. This strategy was adapted for other databases according to their specific syntax requirements, ensuring reproducibility of our systematic search.

### 3.14 Supplementary diagnostic visualizations

Additional visualizations further characterize our findings. The prediction interval visualization (Supplementary Fig. 1) shows the 95 % prediction interval for MBDP incidence ranging from 0.13 to 1.59. While the 95 % CI excluded the null (0.30–0.68, P-value<0.001), the prediction interval includes 1.0, indicating that a future study in a different setting might observe RR > 1 due to heterogeneity. Importantly, the probability of observing a protective effect (RR < 1) in a new study was 97.7 %, providing strong confidence that nutritional prophylaxis is likely beneficial across diverse clinical settings.

The Baujat plot (Supplementary Fig. 2) visualized each study's heterogeneity contribution versus influence on the pooled result. Xu 2024 high-risk contributed 82 % of total Q (Q contribution = 7.99) with moderate influence on the pooled estimate, while Sureshchandra 2024 was most influential due to its largest sample size (n = 150) but contributed minimally to heterogeneity. The remaining studies (Torabi 2014, Natarajan 2014, Xu 2024) clustered in the low-heterogeneity, low-influence quadrant, indicating stable contributions to the pooled effect.

The GOSH plot (Supplementary Fig. 3) displayed all 31 possible subset combinations, including single-study subsets (k = 1) for complete visualization of the effect distribution. This differs from the 26 subsets reported in Supplementary Table 8, which includes only multi-study combinations (k ≥ 2) where heterogeneity statistics can be calculated. The visualization demonstrated clear separation between subset clusters: 15 subsets without Xu 2024 high-risk formed a tight cluster at I^2^ around 0 % with RR around 0.57, representing homogeneous and consistent effects, while 16 subsets including this study spread across I^2^ = 59–88 % with RR ranging from 0.30 to 0.52. This pattern visually confirms Xu 2024 high-risk as the sole driver of observed heterogeneity, with the full model (k = 5) showing I^2^ = 58.9 % and RR = 0.46, pulled toward the stronger effect observed in this high-risk population.

Finally, PET-PEESE regression (Supplementary Fig. 4) estimated bias-corrected effects by extrapolating to SE = 0 (infinite precision). The PET model resulted in bias-corrected RR = 0.80 (β_0_ = −0.222, P-value = 0.54), while PEESE estimated RR = 0.60 (β_0_ = −0.512, P-value = 0.07). The negative regression slope indicated that smaller studies reported stronger effects, consistent with small-study effects or potential publication bias. The non-significant PET intercept (P-value≥0.05) suggests some attenuation of the protective effect after bias correction (naive RR = 0.46 vs. PET-corrected RR = 0.80), however, both bias-corrected estimates continue to indicate benefit, supporting the robustness of our findings.

## 4 Discussion

MBDP represents a significant complication affecting up to 30 % of very low birth weight infants, with high risk of serious consequences including decreased bone mineralization, increased fracture risk, and impaired growth. MBDP originates from interruption of the normal in-utero bone mineralization process, which primarily occurs during the third trimester when calcium and phosphorus accretion rates peak. Premature birth disrupts this significant phase, leaving the underdeveloped skeletal system vulnerable to multiple postnatal factors that further impact bone health [6,[33], [34], [35], [36], [37], [38], [39], [40], [41]].

Despite MBDP clinical significance, the evidence base for its prevention and management has remained limited, with various interventional management strategies studied in isolation and limited synthesis of comparative effectiveness [37,42,43]. Our systematic review and meta-analysis aimed to address this gap by evaluating nutritional, mechanical, and pharmacological interventions for MBDP, with attention to efficacy outcomes, safety profiles, and identification of research gaps regarding the orthopedic management.

We found that the mechanical interventions consistently demonstrated significant benefits for bone mineralization across different measurement modalities. Passive range-of-motion exercises improved bone mineral content by 28 % compared to standard care, while also attenuating the expected physiological decline in quantitative ultrasound speed-of-sound measurements. This suggests that simple, low-cost physical therapy protocols can effectively counteract the detrimental effects of mechanical unloading in the NICU environment. The observed dose-response relationship, with twice-daily protocols showing superior benefits to once-daily regimens, provides practical guidance for better clinical implementation.

Nutritional prophylaxis emerged as a powerful preventive strategy, reducing MBDP incidence by 54 % overall (RR = 0.46, 95 % CI 0.30–0.68), with early calcium/phosphorus supplementation showing particularly high efficacy with 74–84 % reduction in MBDP diagnosis. These findings highlight the importance of mineral supplementation beginning in the first days of life, rather than waiting for biochemical evidence of deficiency to develop. Notably, one study reported complete elimination of severe MBDP (Grade 2–3) with optimized parenteral nutrition formulations containing organic phosphate; although this finding is based on a single study and requires confirmation in larger trials, it suggests that severe manifestations of this condition may be largely preventable with appropriate nutritional strategies.

Weight gain outcomes revealed an interesting pattern across intervention types. Mechanical interventions consistently improved weight gain by 28–31 % compared to controls which challenges the concept that physical activity increases energy expenditure and may interfere with weight gain in premature infants [44,45]. Instead, our findings suggest that passive movement promotes overall metabolic health and growth. In contrast, nutritional interventions showed more variable effects on short-term weight gain, with benefits becoming more observable in longer-term follow-up, as evidenced by significantly higher weights at six-months corrected age with early calcium/phosphorus supplementation.

The biochemical marker findings provide important insights into the underlying mechanisms of intervention effects. Mechanical interventions consistently reduced alkaline phosphatase levels, suggesting decreased bone turnover and improved mineralization. Nutritional interventions, especially those providing calcium/phosphorus supplementation, consistently increased serum phosphate levels. These biochemical observations align with and support the clinically measured improvements in bone mineralization.

Our safety profile analysis offers reassurance for better implementation of both nutritional and mechanical strategies. Nutritional interventions occasionally caused mild biochemical abnormalities, including hypercalcemia, hypercalciuria, and nephrocalcinosis, but these were generally self-limiting or manageable with dose adjustments. The excellent safety profile of mechanical interventions, with no reported adverse events across all studies, is of significant clinical importance. The confirmation that passive movement protocols were well-tolerated, with consistently low pain scores, addresses a common concern about possible discomfort associated with physical therapy in fragile premature infants.

Despite the clinical relevance of fractures in MBDP, only four studies reported fracture outcomes, with none addressing surgical interventions or standardized fracture management protocols. The limited available data suggested a preventive effect of early calcium/phosphorus supplementation on femur fractures in extremely preterm infants, highlighting the importance of prophylactic management. The finding that long-term follow-up showed no difference in lifetime fracture rates between former preterm and term infants suggests that the increased fracture risk associated with MBDP may be transient, resolving with appropriate intervention and ongoing development.

Our findings build upon and extend previous evidence in several important ways. Earlier studies and reviews have suggested benefits of individual interventions, but our meta-analysis provides more validated and precise evidence for efficacy and compares different approaches [46,47]. The 28 % improvement in bone mineralization with mechanical interventions exceeds the effects reported in previous smaller studies, while our finding of a 54 % reduction in MBDP incidence with nutritional prophylaxis represents a more precise estimate than previously available. Bayesian meta-analysis corroborated these findings, with posterior probability of benefit reaching 100 % across all prior specifications, and the NNT of five at typical VLBW baseline risk (40 %) demonstrates strong clinical utility. Our results also provide more comprehensive safety data than prior reviews, facilitating risk-benefit assessments in clinical decision-making.

Despite these strengths, our study has several important limitations that must be acknowledged. First, the included studies had considerable heterogeneity in intervention protocols, limiting our ability to identify optimal dosing or implementation strategies. The nutritional interventions varied widely in calcium/phosphorus dosing, timing, and duration, while mechanical protocols ranged from simple passive movements to more complex resistance exercises. However, advanced heterogeneity analyses (GOSH and Baujat plots) revealed that the observed statistical heterogeneity (I^2^ = 59 %) was driven almost entirely by a single study (Xu 2024 high-risk, contributing 82 % of total Q), reflecting clinically meaningful differences in baseline population risk rather than methodological inconsistency; excluding this study eliminated heterogeneity (I^2^ = 0 %) while maintaining significant protective effects (RR = 0.57). Second, the diagnostic criteria for MBDP varied across studies, with some using biochemical markers, others using radiographic findings, and still others using bone densitometry techniques. This inconsistency complicates the interpretation of MBDP incidence outcomes.

Third, publication bias assessment revealed evidence of small-study effects (Egger's test P-value = 0.028), though trim-and-fill adjusted estimates remained statistically significant (RR = 0.56, 95 % CI 0.38–0.71), and the E-value of 3.26 indicates that an unmeasured confounder would need a significant association with both exposure and outcome to explain away the observed effect. Fourth, the limited data on fracture outcomes represents a significant gap, reflecting both the relatively uncommon nature of fractures even in at-risk populations and methodological challenges in fracture assessment. The complete absence of data on surgical management of MBDP-related complications constitutes a major knowledge gap. Fifth, most studies had relatively short follow-up periods, limiting our understanding of long-term outcomes. Additionally, the risk of bias assessment revealed methodological limitations in many included studies, including inability to blind mechanical interventions, small sample sizes (11 studies with <50 participants), and potential confounding from concurrent therapies.

Based on our findings and identified limitations, we offer several recommendations for future research. First, there is a need for standardized diagnostic criteria and monitoring protocols for MBDP to facilitate consistent assessment and comparison across studies. Second, future trials should evaluate combined intervention protocols that integrate nutritional and mechanical strategies, given their mechanisms of action. Third, long-term follow-up studies are needed to assess the persistence of benefits and any late effects on skeletal development and fracture risk. Fourth, the development and evaluation of standardized fracture management protocols for MBDP-related fractures. Finally, RCTs addressing orthopedic interventions for MBDP-related complications would fill a significant gap in the current evidence base.

## 5 Conclusions

Nutritional prophylaxis was found to reduce MBDP incidence by 54 % (RR = 0.46, 95 % CI 0.30–0.68), with TSA confirming this evidence as conclusive. Early calcium/phosphorus supplementation achieves up to 84 % reduction in high-risk populations, with a NNT of five at typical baseline risk. Mechanical interventions improve bone mineralization by 28 % and weight gain by 5.11 g/kg/day with no adverse events. Both strategies demonstrate favorable safety profiles. Although no studies directly evaluated combined nutritional and mechanical approaches, their complementary mechanisms addressing mineral deficiency and mechanical unloading suggest potential synergistic benefits warranting investigation. Critical evidence gaps persist: no surgical intervention data, no standardized fracture protocols, and absence of combined intervention trials. Future studies are warranted to prioritize combined nutritional-mechanical protocols, standardized MBDP diagnostic criteria, and orthopedic management guidelines for this vulnerable population.

## CRediT authorship contribution statement

**Yazan Jumah Alalwani:** Writing – review & editing, Writing – original draft, Visualization, Methodology, Formal analysis, Conceptualization. **Mujood Ali Alatta:** Writing – original draft, Validation, Methodology, Formal analysis, Conceptualization. **Khawlah Abdullah Almana:** Writing – original draft, Validation, Data curation, Conceptualization. **Amal Alomari:** Writing – original draft, Validation, Methodology, Formal analysis, Conceptualization. **Reem Hameed Alshammari:** Writing – original draft, Project administration, Conceptualization. **Ghadi Naif Alsharif:** Writing – original draft, Visualization, Formal analysis. **Asma Mossa Soweedi:** Writing – original draft, Supervision, Formal analysis. **Sofia Sharif Abualmeza:** Validation, Methodology. **Sara Abdulrahman Alharbi:** Writing – review & editing, Methodology, Formal analysis. **Marwa Abdullrahman Maghrabi:** Writing – review & editing, Visualization, Formal analysis. **Kholoud Khalid Alshehri:** Writing – review & editing, Supervision. **Abdullah Alburaey:** Writing – review & editing, Supervision, Conceptualization. **Ahmed Y. Azzam:** Writing – review & editing, Writing – original draft, Visualization, Validation, Conceptualization.

## Consent to participate

Not applicable.

## Ethical approval

Institutional Review Board (IRB) approval was not required for this systematic review and meta-analysis as it included analysis of previously published data.

## Funding

This study received no specific grant from any funding agency in the public, commercial, or not-for-profit sectors.

## Conflict of interest

The authors declare that they have no conflict of interest.
