# Supplementary material for: Interventions for metabolic bone disease of prematurity: A systematic review and meta-analysis
Source: Metabol Open. 2026 Jan 19;29:100445. doi: 10.1016/j.metop.2026.100445 (PMC12858363; doi:10.1016/j.metop.2026.100445)
Supplement: Multimedia component 1 [file mmc1.pdf]

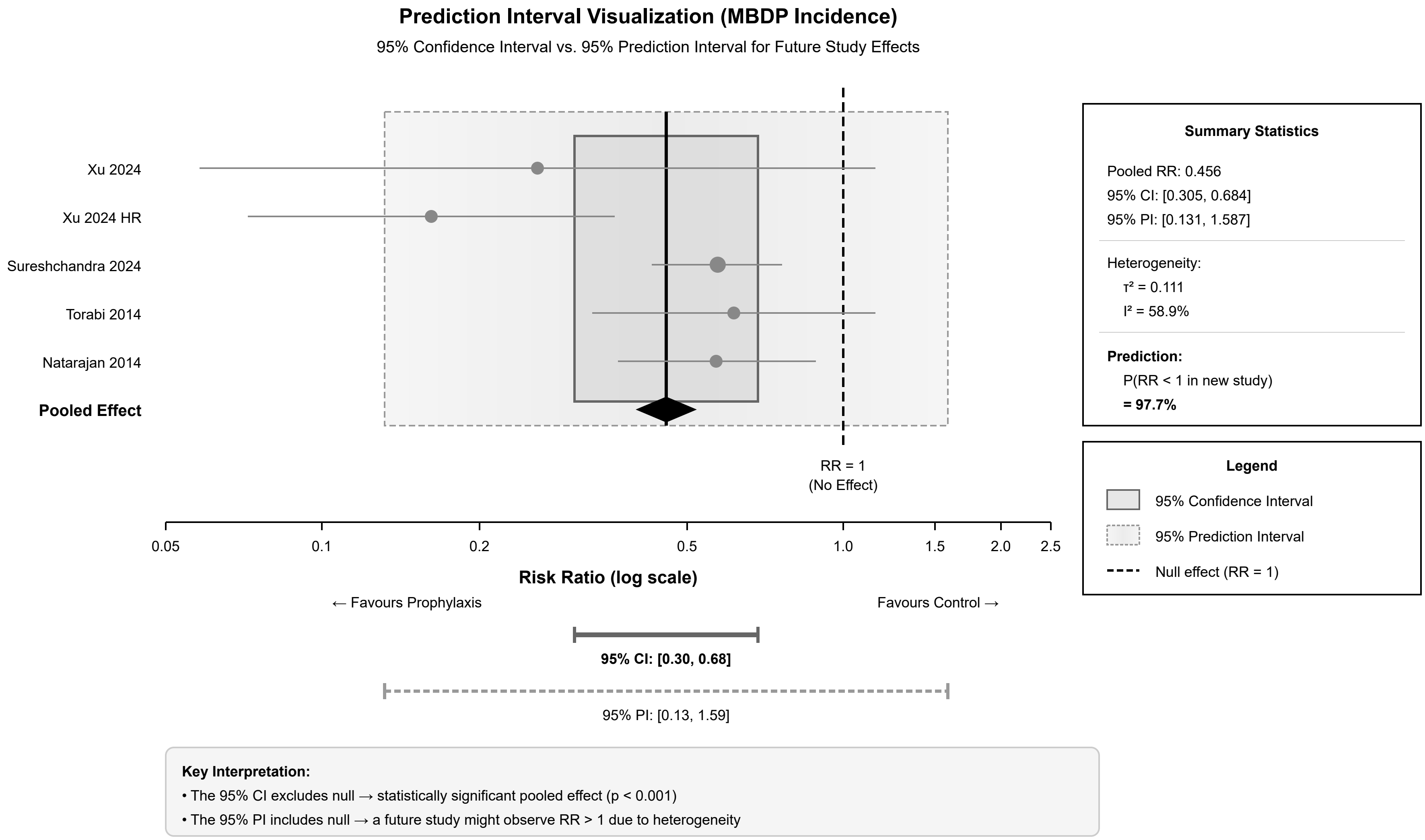
Prediction Interval Visualization (MBDP Incidence)
95% Confidence Interval vs. 95% Prediction Interval for Future Study Effects
Xu 2024
Xu 2024 HR
Sureshchandra 2024
Torabi 2014
Natarajan 2014
Pooled Effect
RR = 1
(No Effect)
0.05
0.1
0.2
0.5
1.0
1.5
2.0
2.5
Risk Ratio (log scale)
← Favours Prophylaxis
Favours Control →
95% CI: [0.30, 0.68]
95% PI: [0.13, 1.59]
Summary Statistics
Pooled RR: 0.456
95% CI: [0.305, 0.684]
95% PI: [0.131, 1.587]
Heterogeneity:
τ² = 0.111
I² = 58.9%
Prediction:
P(RR < 1 in new study)
= 97.7%
Legend
95% Confidence Interval
95% Prediction Interval
Null effect (RR = 1)
Key Interpretation:
• The 95% CI excludes null → statistically significant pooled effect (p < 0.001)
• The 95% PI includes null → a future study might observe RR > 1 due to heterogeneity
