# Supplementary material for: Interventions for metabolic bone disease of prematurity: A systematic review and meta-analysis
Source: Metabol Open. 2026 Jan 19;29:100445. doi: 10.1016/j.metop.2026.100445 (PMC12858363; doi:10.1016/j.metop.2026.100445)
Supplement: Multimedia component 2 [file mmc2.pdf]

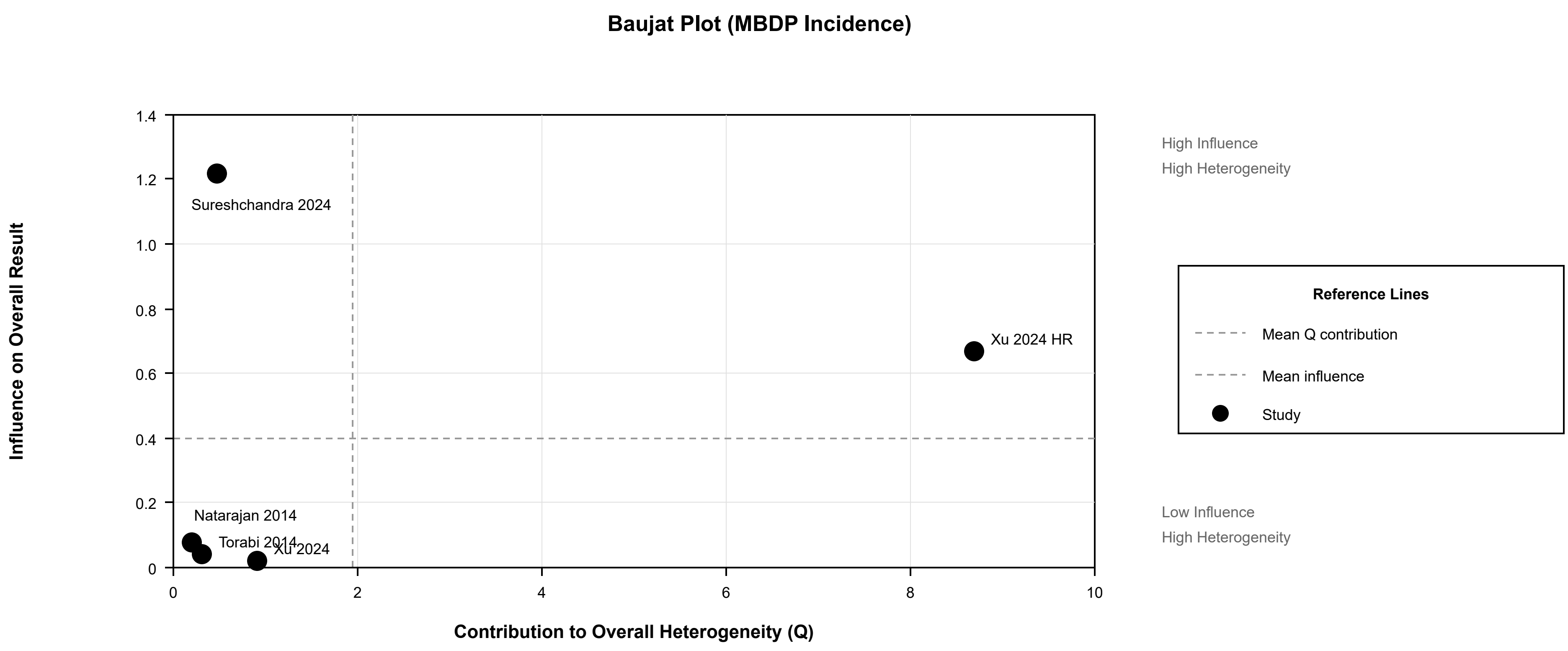

Interpretation: Studies in the upper-right quadrant contribute most to heterogeneity AND influence the pooled result substantially.

- Xu 2024 HR: Primary source of heterogeneity (Q contribution = 7.99, 82% of total Q), with moderate influence on pooled result.
- Sureshchandra 2024: Most influential on pooled result due to largest sample size (n=150), but low contribution to heterogeneity.
- Remaining studies (Torabi 2014, Natarajan 2014, Xu 2024) cluster in lower-left: minimal heterogeneity contribution and low influence.
