# Supplementary material for: Interventions for metabolic bone disease of prematurity: A systematic review and meta-analysis
Source: Metabol Open. 2026 Jan 19;29:100445. doi: 10.1016/j.metop.2026.100445 (PMC12858363; doi:10.1016/j.metop.2026.100445)
Supplement: Multimedia component 3 [file mmc3.pdf]

GOSH Plot (MBDP Incidence)

Graphical Overview for Study Heterogeneity — All 31 Subset Combinations

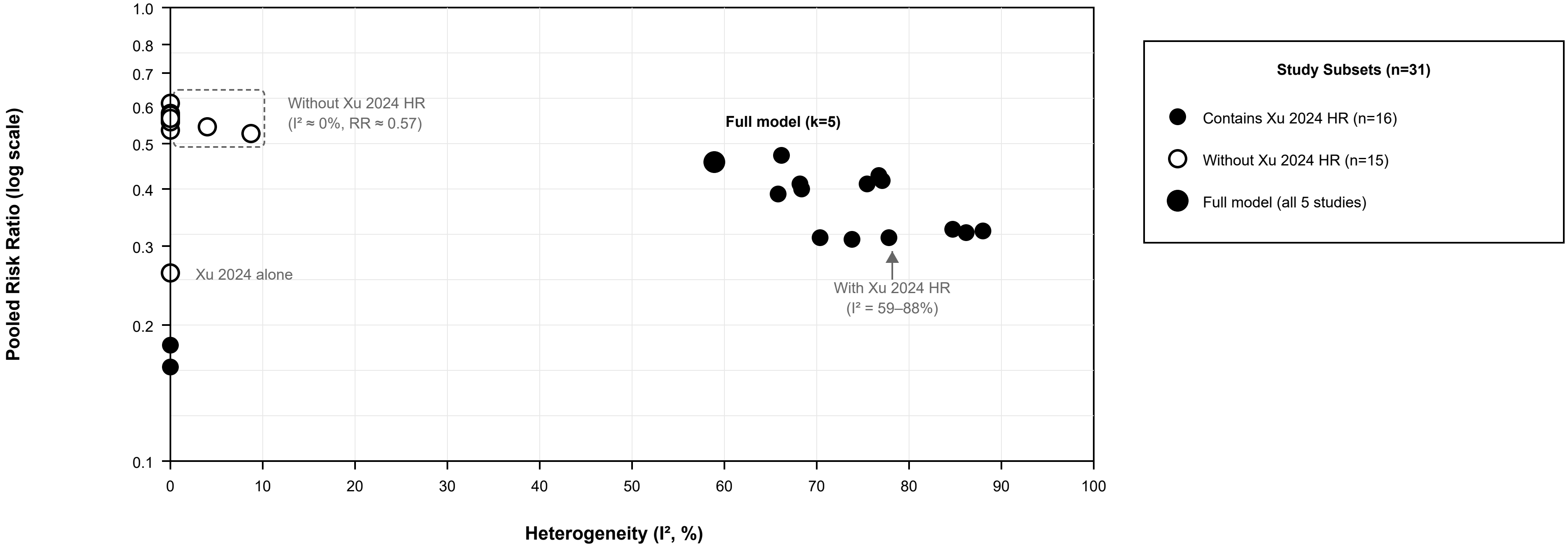

Interpretation: Each point represents a unique subset of studies with its pooled effect (Y-axis) and heterogeneity (X-axis).
The clear separation demonstrates that Xu 2024 HR is the sole driver of heterogeneity:

- Subsets WITHOUT Xu 2024 HR (open circles): Cluster at $I^2 \approx 0\%$ with RR ≈ 0.57 — homogeneous and consistent.
- Subsets WITH Xu 2024 HR (filled circles): Show I^2 from 59% to 88% — this study introduces all observed heterogeneity.
- The full model (k=5) shows $I^2 = 58.9\%$ and RR = 0.46, pulled toward Xu 2024 HR's stronger effect (RR = 0.16).
