# Supplementary material for: Interventions for metabolic bone disease of prematurity: A systematic review and meta-analysis
Source: Metabol Open. 2026 Jan 19;29:100445. doi: 10.1016/j.metop.2026.100445 (PMC12858363; doi:10.1016/j.metop.2026.100445)
Supplement: Multimedia component 4 [file mmc4.pdf]

PET-PEESE Regression Analysis (MBDP Incidence)

Precision-Effect Test and Precision-Effect Estimate with Standard Error

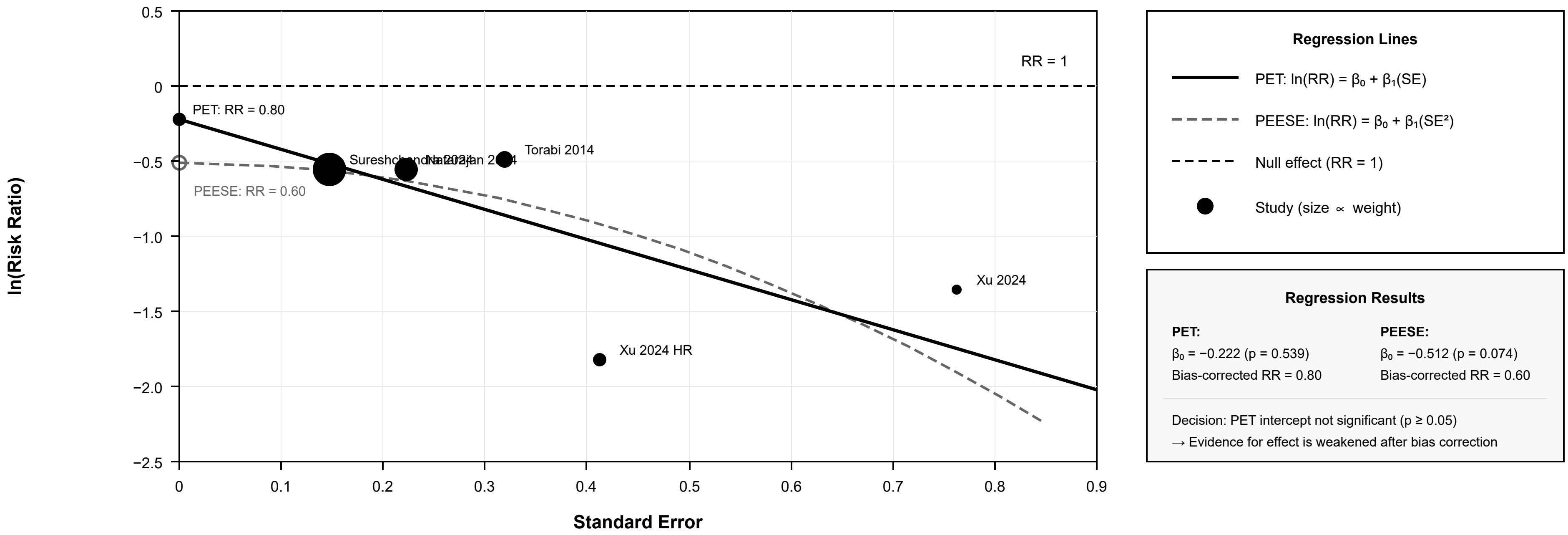

Interpretation: PET-PEESE regression extrapolates effects to SE=0 (infinite precision) to estimate bias-corrected treatment effect.

- The negative slope indicates smaller studies (higher SE) report stronger effects — consistent with publication bias or small-study effects.
- Naive pooled effect: RR = 0.46. PET bias-corrected: RR = 0.80. PEESE bias-corrected: RR = 0.60.
- PET intercept is non-significant ($p = 0.54$), suggesting the protective effect may be partially attributable to publication bias.
- Xu 2024 HR drives the regression slope due to its strong effect (RR = 0.16) combined with moderate SE — key outlier in bias assessment.
