# Supplementary material for: Interventions for metabolic bone disease of prematurity: A systematic review and meta-analysis
Source: Metabol Open. 2026 Jan 19;29:100445. doi: 10.1016/j.metop.2026.100445 (PMC12858363; doi:10.1016/j.metop.2026.100445)
Supplement: Multimedia component 5 [file mmc5.docx]

**Supplementary Table 1:** Risk of Bias Assessment.

| **Study** | **Year** | **Design** | **Randomization** | **Allocation Concealment** | **Blinding** | **Outcome Assessment** | **Incomplete Data** | **Reporting** | **Other Bias** | **Overall Risk** |
| --- | --- | --- | --- | --- | --- | --- | --- | --- | --- | --- |
| Xu et al. | 2024 | Retrospective | N/A | N/A | N/A | Low | Low | Low | Moderate confounding | Moderate |
| Sureshchandra et al. | 2024 | Retrospective | N/A | N/A | N/A | Low | Low | Low | Moderate confounding | Moderate |
| Krithika et al. | 2022 | QI Initiative | N/A | N/A | N/A | Low | Moderate | Low | Serious confounding | Serious |
| Torró-Ferrero et al. | 2022 | RCT | Low | Low | High (unblinded) | Low | Low | Low | Low | Moderate |
| Kołodziejczyk-Nowotarska et al. | 2021 | Pragmatic RCT | Low | Low | High (unblinded) | Low | Low | Low | Low | Moderate |
| Litmanovitz et al. | 2016 | RCT | Low | Low | Low | Low | Low | Low | Low | Low |
| Rustico et al. | 2015 | Retrospective | N/A | N/A | N/A | Low | Moderate | Low | Serious confounding | Serious |
| Torabi et al. | 2014 | RCT | Low | Low | High (unblinded) | Low | Low | Low | Low | Low |
| Natarajan et al. | 2014 | Double-blind RCT | Low | Low | Low | Low | Low | Low | Low | Low |
| Vignochi et al. | 2012 | RCT | Low | Low | High (unblinded) | Low | Low | Low | Low | Moderate |
| Moyer-Mileur et al. | 2008 | RCT | Low | Low | High (unblinded) | Low | Low | Low | Low | Moderate |
| Vignochi et al. | 2008 | RCT | Low | Low | High (unblinded) | Low | Low | Low | Low | Moderate |
| Litmanovitz et al. | 2007 | RCT | Low | Low | Low | Low | Low | Low | Low | Low |
| Nemet et al. | 2002 | RCT | Low | Low | High (unblinded) | Low | Low | Low | Low | Low |
| Moyer-Mileur et al. | 2000 | RCT | Low | Low | High (unblinded) | Low | Low | Low | Low | Low |
| Fewtrell et al. | 1999 | RCT | Low | Low | High (unblinded) | Low | Low | Low | Low | Moderate |
| Moyer-Mileur et al. | 1995 | Pilot RCT | Low | Unclear | High (unblinded) | Low | Low | Low | Low | Moderate |

***Notes:*** *Risk of bias assessed using ROB-2 for RCTs and ROBINS-I for non-randomized studies. N/A = Not applicable for non-randomized designs. QI = Quality Improvement. Overall risk classified as Low, Moderate, or Serious based on domain assessments.*

**Supplementary Table 2:** Intervention Protocols and Fracture Data.

| **Study** | **Year** | **Intervention Type** | **Protocol Description** | **Duration** | **Fracture Incidence** | **Fracture Management** |
| --- | --- | --- | --- | --- | --- | --- |
| Xu et al. | 2024 | Nutritional | Early Ca/P (IV→enteral), Ca 65-100 mg/kg/d, P 35-60 mg/kg/d | Throughout NICU stay | 0/102 vs 3/132 (2.27%) | Healing at 6 months |
| Sureshchandra et al. | 2024 | Nutritional | Enhanced PN: Ca 2.3, P 1.8 mmol/kg/d, organic phosphate | Variable | 0/57 vs 1/93 (1%) | NR |
| Krithika et al. | 2022 | Nutritional | Sequential: early HMF, parenteral Ca/P, higher targets | Throughout NICU stay | NR | NA |
| Torró-Ferrero et al. | 2022 | Mechanical | RLT or passive movements 15-16 min/day, 5 days/week | 4 weeks | NR | NA |
| Kołodziejczyk-Nowotarska et al. | 2021 | Nutritional | Monitored vitamin D dosing based on 25(OH)D levels | Variable | NR | NA |
| Litmanovitz et al. | 2016 | Mechanical | Passive ROM exercises once vs twice daily | 8 weeks | NR | NA |
| Rustico et al. | 2015 | Pharmacological | Calcitriol 0.05-0.08 μg/kg/d | Median 207 days | 53% had fractures at baseline | Managed with calcitriol |
| Torabi et al. | 2014 | Nutritional | Elemental Ca 45 mg/kg/d + P 24 mg/kg/d + Vit D 400 IU/d | 4 weeks | NR | NA |
| Natarajan et al. | 2014 | Nutritional | Vitamin D 800 IU vs 400 IU daily | Until discharge | NR | NA |
| Vignochi et al. | 2012 | Mechanical | Passive movements + joint compression, 15 min/day, 5d/wk | 4 weeks | NR | NA |
| Moyer-Mileur et al. | 2008 | Mechanical | Mother or therapist ROM exercises | 4 weeks | NR | NA |
| Vignochi et al. | 2008 | Mechanical | Passive ROM + respiratory movements, 15 min/day, 5d/wk | 4 weeks | NR | NA |
| Litmanovitz et al. | 2007 | Mechanical | Passive ROM exercises 10 min/day, 5 days/week | 8 weeks | NR | NA |
| Nemet et al. | 2002 | Mechanical | Passive ROM + gentle compression, 5-10 min/day, 5d/wk | 4 weeks | NR | NA |
| Moyer-Mileur et al. | 2000 | Mechanical | Passive ROM + gentle compression, 5-10 min/day, 5d/wk | ~4 weeks | NR | NA |
| Fewtrell et al. | 1999 | Nutritional | Neonatal diet variations (BBM vs PTF vs TF) | Neonatal period | No difference at 8-12 years | Long-term follow-up |
| Moyer-Mileur et al. | 1995 | Mechanical | Passive ROM with resistance, 5-10 min/day, 5d/wk | 4 weeks | NR | NA |

***Abbreviations:*** *BBM, banked breast milk; Ca, calcium; HMF, human milk fortifier; NA, not assessed; NICU, neonatal intensive care unit; NR, not reported; P, phosphorus; PN, parenteral nutrition; PTF, preterm formula; RLT, reflex locomotion therapy; ROM, range of motion; TF, term formula; Vit D, vitamin D.*

**Supplementary Table 3:** Adverse Events and Safety Profile.

| **Study** | **Year** | **Intervention Type** | **Intervention** | **Adverse Event** | **Frequency** | **Severity** | **Management** |
| --- | --- | --- | --- | --- | --- | --- | --- |
| Sureshchandra et al. | 2024 | Nutritional | Enhanced PN | Hypocalcemia | 0% vs 9% (control) | Mild | None required |
| Sureshchandra et al. | 2024 | Nutritional | Enhanced PN | Hypercalcemia | 38% vs 19% (control) | Mild | None required |
| Sureshchandra et al. | 2024 | Nutritional | Enhanced PN | Hypophosphatemia | 25% vs 51% (control) | Mild | None required |
| Kołodziejczyk-Nowotarska et al. | 2021 | Nutritional | Vitamin D | Hypercalcemia | 5% | Mild | Dose adjustment |
| Kołodziejczyk-Nowotarska et al. | 2021 | Nutritional | Vitamin D | Hypercalciuria | 20-28% | Mild | Monitoring |
| Kołodziejczyk-Nowotarska et al. | 2021 | Nutritional | Vitamin D | Vitamin D excess | 2-24% | Mild | Dose adjustment |
| Kołodziejczyk-Nowotarska et al. | 2021 | Nutritional | Vitamin D | Nephrocalcinosis | 8-28% | Moderate | Monitoring |
| Rustico et al. | 2015 | Pharmacological | Calcitriol | Hypercalcemia | Occasional | Mild | Dose adjustment |
| Rustico et al. | 2015 | Pharmacological | Calcitriol | Hypercalciuria | Occasional | Mild | Monitoring |
| Krithika et al. | 2022 | Nutritional | PN formulation | TPN precipitation | 2 incidents | Moderate | Protocol modification |
| Torró-Ferrero et al. | 2022 | Mechanical | RLT/Passive | None reported | 0% | N/A | N/A |
| Litmanovitz et al. | 2016 | Mechanical | Passive ROM | None reported | 0% | N/A | N/A |
| Vignochi et al. | 2012 | Mechanical | Passive ROM | Pain (NIPS score) | 0-2 (low) | Minimal | None required |
| Vignochi et al. | 2008 | Mechanical | Passive ROM | None reported | 0% | N/A | N/A |
| Litmanovitz et al. | 2007 | Mechanical | Passive ROM | None reported | 0% | N/A | N/A |
| Nemet et al. | 2002 | Mechanical | Passive ROM | None reported | 0% | N/A | N/A |
| Moyer-Mileur et al. | 2000 | Mechanical | Passive ROM | None reported | 0% | N/A | N/A |
| Moyer-Mileur et al. | 2008 | Mechanical | ROM exercises | None reported | 0% | N/A | N/A |
| Moyer-Mileur et al. | 1995 | Mechanical | Passive ROM | None reported | 0% | N/A | N/A |
| Natarajan et al. | 2014 | Nutritional | Vitamin D | None significant | NR | N/A | N/A |
| Torabi et al. | 2014 | Nutritional | Ca/P supplementation | None significant | NR | N/A | N/A |

***Abbreviations:*** *Ca/P, calcium/phosphorus; N/A, not applicable; NIPS, Neonatal Infant Pain Scale; NR, not reported; PN, parenteral nutrition; RLT, reflex locomotion therapy; ROM, range of motion; TPN, total parenteral nutrition.*

**Supplementary Table 4:** Research Gaps and Clinical Recommendations.

| **Domain** | **Study Evidence** | **Findings/Identified Gap** | **Clinical Significance** | **Recommendation** |
| --- | --- | --- | --- | --- |
| Growth: Length | Xu 2024 | PSG: 68.5±3.9 vs Non-PSG: 64.1±3.2 cm at 6mo | Early Ca/P improves length | Implement early Ca/P supplementation |
|  | Vignochi 2008 | PG: 1.3±0.3 vs CG: 0.8±0.2 cm/wk | Mechanical stimulation improves growth | Consider passive movement therapy |
|  | Fewtrell 1999 | Preterm shorter vs term at 8-12 years | Persistent growth disadvantage | Long-term growth monitoring needed |
| Growth: Head Circ. | Xu 2024 | PSG: 44.1±1.8 vs Non-PSG: 42.5±2.1 cm | Improved head growth with intervention | Monitor head circumference closely |
| Fracture Prevention | Xu 2024 | 0% vs 2.27% fractures | Prophylaxis may prevent fractures | Early supplementation in high-risk infants |
| Fracture Management | All studies | No surgical intervention data | CRITICAL GAP | RCTs needed for fracture management |
| Orthopedic Outcomes | All studies | No deformity assessment data | CRITICAL GAP | Standardized skeletal assessment needed |
| Long-term Outcomes | Fewtrell 1999 | No fracture difference at 8-12 years | MBDP effects may be transient | Long-term follow-up studies needed |
| Combined Interventions | None | No studies of combined nutritional + mechanical | SIGNIFICANT GAP | Trials of combined approaches needed |
| Diagnostic Criteria | Variable | Heterogeneous definitions across studies | Limits comparability | Standardized MBDP criteria needed |
| Biomarkers | Multiple | ALP thresholds vary (500-900 U/L) | Inconsistent screening | Consensus on biochemical thresholds |
| Dosing Optimization | Variable | Ca/P doses vary widely | Optimal dosing unknown | Dose-finding studies needed |
| Timing of Intervention | Xu 2024, Sureshchandra 2024 | Early intervention more effective | First days of life critical | Start prophylaxis within 48-72 hours |
| Mechanical Protocol | Multiple | Protocols vary (5-16 min, 1-2×/day) | Optimal protocol unknown | Standardization of PT protocols needed |

***Abbreviations:*** *Ca/P, calcium/phosphorus; CG, control group; Head Circ., head circumference; MBDP, metabolic bone disease of prematurity; PG, physiotherapy group; PSG, prophylactic supplementation group; PT, physical therapy; RCT, randomized controlled trial.*

**Supplementary Table 5:** Heterogeneity Estimator Comparison for MBDP Incidence.

| **Estimator** | **τ²** | **τ** | **I²** | **RR** | **95% CI** | **p-value** | **Notes** |
| --- | --- | --- | --- | --- | --- | --- | --- |
| DerSimonian-Laird | 0.111 | 0.333 | 64.0% | 0.456 | 0.305–0.684 | <0.001 | Traditional method |
| **REML (Primary)** | **0.000** | **0.000** | **58.9%** | **0.519** | **0.419–0.644** | **<0.001** | **Cochrane 2024 recommended** |
| Paule-Mandel | 0.202 | 0.449 | 67.0% | 0.439 | 0.267–0.719 | 0.001 | Iterative estimator |
| Sidik-Jonkman | 0.202 | 0.449 | 67.0% | 0.439 | 0.267–0.719 | 0.001 | Alternative iterative |
| Maximum Likelihood | 0.062 | 0.248 | 61.7% | 0.473 | 0.337–0.666 | <0.001 | ML-based |
| Hunter-Schmidt | 0.057 | 0.238 | 61.4% | 0.476 | 0.341–0.664 | <0.001 | Weighted variance |
| Q-Profile CI for τ² | — | — | — | — | 0.000–0.179 | — | 95% CI for between-study variance |

***Notes:*** *REML (Restricted Maximum Likelihood) selected as primary estimator per Cochrane Handbook 2024 recommendations. All estimators demonstrate significant protective effect (all p<0.01). τ² = between-study variance; τ = between-study standard deviation; I² = percentage of variability due to heterogeneity; RR = risk ratio; CI = confidence interval.*

**Supplementary Table 6:** Leave-One-Out Sensitivity Analysis for MBDP Incidence.

| **Study Excluded** | **RR** | **95% CI** | **P-value** | **I²** | **τ²** | **Conclusion Change?** |
| --- | --- | --- | --- | --- | --- | --- |
| None (Full Analysis) | 0.519 | 0.419–0.644 | <0.001 | 58.9% | 0.000 | — (Reference) |
| Xu 2024 (MBDP) | 0.51 | 0.41–0.64 | <0.001 | 66.2% | 0.021 | No |
| **Xu 2024 (High Risk)** | **0.57** | **0.47–0.69** | **<0.001** | **0.0%** | **0.000** | **No; I² eliminated** |
| Sureshchandra 2024 | 0.46 | 0.30–0.71 | <0.001 | 65.8% | 0.098 | No |
| Torabi 2014 | 0.49 | 0.38–0.64 | <0.001 | 68.1% | 0.032 | No |
| Natarajan 2014 | 0.47 | 0.35–0.64 | <0.001 | 68.4% | 0.044 | No |

***Notes:*** *Leave-one-out analysis demonstrates robustness of findings. All iterations maintain statistical significance (p<0.001). Excluding "Xu 2024 High Risk" subgroup eliminates heterogeneity (I² drops from 58.9% to 0.0%), identifying this study as the primary heterogeneity driver. Abbreviations: CI, confidence interval; RR, risk ratio.*

**Supplementary Table 7:** Influence Diagnostics for MBDP Incidence Meta-Analysis.

| **Study** | **Weight (%)** | **DFBETAS** | **Cook's D** | **Hat Value** | **Studentized Residual** | **Outlier Status** | **Influence** |
| --- | --- | --- | --- | --- | --- | --- | --- |
| Xu 2024 (MBDP) | 5.2% | -0.132 | 0.018 | 0.021 | -0.89 | No | Low |
| Xu 2024 (High Risk) | 18.2% | -0.778 | 0.651 | 0.070 | -2.31 | Borderline | Moderate |
| Sureshchandra 2024 | 42.1% | 0.729 | 1.183 | 0.551 | 1.42 | **HIGH LEVERAGE** | High |
| Torabi 2014 | 14.2% | 0.194 | 0.043 | 0.118 | 0.58 | No | Low |
| Natarajan 2014 | 20.3% | 0.241 | 0.077 | 0.240 | 0.64 | No | Low |
| **THRESHOLDS** | **—** | **>1.0** | **>0.80** | **>0.40** | **>\|2.5\|** | **—** | **—** |

***Notes:*** *DFBETAS measures change in pooled estimate when study removed (threshold >1.0 = influential). Cook's Distance measures overall influence on model fit (threshold >4/k = 0.80 for k=5 studies). Hat value measures leverage in analysis (threshold >2/k = 0.40). Sureshchandra 2024 shows high leverage due to large sample size and weight. Xu 2024 High Risk shows borderline influence on heterogeneity.*

**Supplementary Table 8:** GOSH (Graphical Display of Study Heterogeneity) Analysis.

| **Parameter** | **Value** | **Interpretation** | **Clinical Relevance** |
| --- | --- | --- | --- |
| **All-Subsets Analysis:** | | | |
| Total Subsets Analyzed | 26 (all k≥2 combinations) | — | — |
| RR Range | 0.180 – 0.585 | All protective | Effect consistent |
| RR Mean (SD) | 0.447 (0.113) | — | — |
| RR Median | 0.441 | — | — |
| Subsets with RR < 1 | 26/26 (100%) | Universal protective effect | Robust finding |
| I² Range | 0% – 94.4% | Variable heterogeneity | — |
| Subsets with I² = 0% | 10/26 (38.5%) | Many homogeneous combinations | — |
| **Heterogeneity Drivers:** | | | |
| Excluding Xu 2024 MBDP | Mean I² = 52.9% | Minor impact | — |
| Excluding Xu 2024 High Risk | Mean I² = 1.3% | **Primary Driver** | Key heterogeneity source |
| Excluding Sureshchandra 2024 | Mean I² = 44.0% | Moderate impact | — |
| Excluding Torabi 2014 | Mean I² = 47.1% | Minor impact | — |

***Notes:*** *GOSH analysis evaluates all possible study combinations to assess effect stability and identify heterogeneity sources. All 26 subsets (k≥2) demonstrate protective effect (RR<1), confirming robustness. Excluding Xu 2024 High Risk subgroup reduces mean I² to 1.3%, identifying it as the primary heterogeneity driver, likely due to stringent diagnostic criteria in high-risk population.*

**Supplementary Table 9:** Bayesian Meta-Analysis Results.

| **Analysis** | **Prior Specification** | **Posterior RR** | **95% CrI** | **P(RR<1)** | **P(RR<0.5)** | **P(RR<0.8)** |
| --- | --- | --- | --- | --- | --- | --- |
| Frequentist (REML) | — | 0.519 | 0.419–0.644 | — | — | — |
| **Bayesian (Weakly Informative)** | **log(RR) ~ N(0, 1)** | **0.523** | **0.423–0.648** | **100.0%** | **33.8%** | **100.0%** |
| Bayesian (Skeptical) | log(RR) ~ N(0, 0.5) | 0.535 | 0.434–0.660 | 100.0% | — | 100.0% |
| Bayesian (Enthusiastic) | log(RR) ~ N(log(0.6), 0.5) | 0.523 | 0.424–0.645 | 100.0% | — | 100.0% |
| **INTERPRETATION** |  |  |  |  |  |  |
| P(RR<1) = 100% | — | — | — | Certain benefit | — | — |
| P(RR<0.8) = 100% | — | — | — | — | — | Clinically meaningful |
| P(RR<0.5) = 33.8% | — | — | — | — | 1 in 3 chance of >50% reduction | — |

***Notes:*** *Bayesian analysis provides probability-based inference. Weakly informative prior N(0,1) on log(RR) scale centered at null effect. Skeptical prior assumes smaller effect variance. Enthusiastic prior centered at expected protective effect. Results robust across all prior specifications. CrI = Credible Interval (Bayesian equivalent of confidence interval). P(RR<X) = posterior probability that true effect is less than X.*

**Supplementary Table 10:** Number Needed to Treat (NNT) at Various Baseline Risks.

| **Baseline MBDP Risk** | **Population** | **ARR** | **95% CI** | **NNT** | **95% CI** |
| --- | --- | --- | --- | --- | --- |
| 20% | Low-risk VLBW | 9.6% | 7.1–11.6% | 10.4 | 8.6–14.1 |
| 30% | Moderate-risk VLBW | 14.4% | 10.7–17.4% | 6.9 | 5.7–9.3 |
| 40% | Typical VLBW | 19.2% | 14.2–23.2% | 5.2 | 4.3–7.0 |
| **42.2%** | **Observed CER (this study)** | **20.3%** | **15.0–24.5%** | **4.9** | **4.1–6.6** |
| 50% | High-risk ELBW | 24.0% | 17.8–29.0% | 4.2 | 3.4–5.6 |
| 60% | Very high-risk ELBW | 28.8% | 21.3–34.8% | 3.5 | 2.9–4.7 |
| **At observed baseline (42.2%)** | **—** | **—** | **—** | **Treat 5 to prevent 1 case** | **—** |

***Notes:*** *NNT calculated using pooled RR (0.519) at various baseline risks. ARR = Absolute Risk Reduction = Baseline Risk × (1 - RR). NNT = 1/ARR. Lower NNT indicates greater clinical impact. At the observed control event rate of 42.2%, approximately 5 infants need to receive nutritional prophylaxis to prevent 1 case of MBDP. NNT decreases (more favorable) as baseline risk increases.* ***Abbreviations:*** *ARR, absolute risk reduction; CER, control event rate; CI, confidence interval; ELBW, extremely low birth weight; NNT, number needed to treat; VLBW, very low birth weight.*

**Supplementary Table 11:** Full Electronic Search Strategy (PubMed/MEDLINE).

| **Search Line** | **Search Terms** | **Results** |
| --- | --- | --- |
| #1 | (preterm[tiab] OR premature[tiab] OR "very low birth weight"[tiab] OR VLBW[tiab] OR "extremely low birth weight"[tiab] OR ELBW[tiab] OR "low birth weight"[tiab] OR LBW[tiab]) | — |
| #2 | ("metabolic bone disease"[tiab] OR MBD[tiab] OR osteopenia[tiab] OR rickets[tiab] OR "bone mineralization"[tiab] OR "bone mineral density"[tiab] OR BMD[tiab] OR "bone mineral content"[tiab] OR BMC[tiab]) | — |
| #3 | (calcium[tiab] OR phosphorus[tiab] OR phosphate[tiab] OR "vitamin D"[tiab] OR calcitriol[tiab] OR ergocalciferol[tiab] OR cholecalciferol[tiab]) | — |
| #4 | (exercise[tiab] OR "physical therapy"[tiab] OR "passive movement"[tiab] OR "range of motion"[tiab] OR mechanical[tiab] OR loading[tiab] OR vibration[tiab]) | — |
| #5 | (bisphosphonate[tiab] OR orthopedic[tiab] OR orthotic[tiab] OR casting[tiab] OR surgery[tiab] OR fixation[tiab] OR fracture[tiab]) | — |
| #6 | (prevention[tiab] OR treatment[tiab] OR therapy[tiab] OR intervention[tiab] OR supplementation[tiab]) | — |
| #7 | #3 OR #4 OR #5 OR #6 | — |
| #8 | #1 AND #2 AND #7 | — |
| #9 | #8 AND (English[lang]) | — |
| **#10** | **#9 (Filters: Humans)** | **1,842** |
| **Search Date** | **May 5, 2025; Database inception to search date** | **—** |

***Notes:*** *Search strategy adapted for other databases (Scopus, Cochrane Library, Web of Science, Google Scholar) using equivalent syntax. No date restrictions applied from database inception. Language restricted to English. [tiab] = Title/Abstract field. Duplicate removal performed using reference management software. Final results after deduplication: 1,842 records screened.*
